# Peptidoglycan Endopeptidase PBP7 Facilitates the Recruitment of FtsN to the Divisome and Promotes Peptidoglycan Synthesis in *Escherichia coli*

**DOI:** 10.1111/mmi.15321

**Published:** 2024-09-30

**Authors:** Xinwei Liu, Gabriela Boelter, Waldemar Vollmer, Manuel Banzhaf, Tanneke den Blaauwen

**Affiliations:** ^1^ Bacterial Cell Biology and Physiology, Swammerdam Institute for Life Sciences, Faculty of Science University of Amsterdam Amsterdam The Netherlands; ^2^ Institute of Microbiology & Infection and School of Biosciences University of Birmingham Edgbaston Birmingham UK; ^3^ Centre for Bacterial Cell Biology, Biosciences Institute Faculty of Medical Sciences, Newcastle University, Framlington Place Newcastle upon Tyne UK; ^4^ Institute for Molecular Bioscience The University of Queensland Brisbane Queensland Australia

**Keywords:** divisome, *Escherichia coli*, PBP7, penicillin‐binding proteins, peptidoglycan hydrolases, peptidoglycan synthesis

## Abstract

*Escherichia coli* has many periplasmic hydrolases to degrade and modify peptidoglycan (PG). However, the redundancy of eight PG endopeptidases makes it challenging to define specific roles to individual enzymes. Therefore, the cellular role of PBP7 (encoded by *pbpG*) is not clearly defined. In this work, we show that PBP7 localizes in the lateral cell envelope and at midcell. The C‐terminal α‐helix of PBP7 is crucial for midcell localization but not for its activity, which is dispensable for this localization. Additionally, midcell localization of PBP7 relies on the assembly of FtsZ up to FtsN in the divisome, and on the activity of PBP3. PBP7 was found to affect the assembly timing of FtsZ and FtsN in the divisome. The absence of PBP7 slows down the assembly of FtsN at midcell. The Δ*pbpG* mutant exhibited a weaker incorporation of the fluorescent D‐amino acid HADA, reporting on transpeptidase activity, compared to wild‐type cells. This could indicate reduced PG synthesis at the septum of the Δ*pbpG* strain, explaining the slower accumulation of FtsN and suggesting that endopeptidase‐mediated PG cleavage may be a rate‐limiting step for septal PG synthesis.

## Graphical abstract

The peptidoglycan (PG) hydrolase PBP7 localizes to both the lateral cell envelope and midcell in *Escherichia coli*, and its midcell localization is dependent on an active divisome. PBP7 influences the timing of FtsZ and FtsN assembly within the divisome. In the absence of PBP7, FtsN assembly is slower and septal PG synthesis is reduced in the absence of PBP7. This regulation of divisome activity by PBP7 may arise from its space‐making activity.

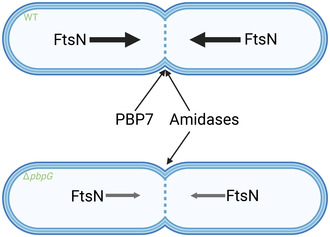

## 1 Introduction

Peptidoglycan (PG) is a mesh‐like component of the bacterial cell envelope that is critical for maintaining cell shape and providing protection against high intracellular turgor. Structurally, PG is composed of linear glycan strands that are bridged by short‐peptide chains. The glycan strands are made up of alternating *N*‐acetylmuramic acid (MurNAc) and *N*‐acetylglucosamine (GlcNAc) disaccharide units, which are linked together by β‐1,4 glycosidic bonds (Vollmer, Blanot, and de Pedro 2008). The length of the glycan strands can vary, ranging from 2 to ≥ 30 disaccharide units in *Escherichia coli* (Glauner, Höltje, and Schwarz 1988; Harz, Burgdorf, and Höltje 1990). A short peptide made of 5 amino‐acid residues is covalently attached to the MurNAc (Glauner, Höltje, and Schwarz 1988). In *E. coli*, the pentapeptide is composed of L‐alanine, D‐glutamic acid, meso‐diaminopimelic acid (meso‐DAP), D‐alanine, and D‐alanine. About 40% of the peptides are cross‐linked to adjacent peptides. About 90% of cross‐links are produced by DD‐transpeptidases between D‐alanine at position 4 of one peptide and meso‐DAP at position 3 of the other (D‐Ala−mDAP, or 4–3) and about 10% are between two meso‐mDAP residues (mDAP−mDAP, or 3–3) introduced by the LD‐transpeptidases (LDTs) LdtD and LdtE (Glauner, Höltje, and Schwarz 1988; Mainardi et al. 2008; Pisabarro, de Pedro, and Vázquez 1985).

PG exhibits both rigid and dynamic properties. Within one cell cycle, up to half of the pre‐existing PG is degraded and the released turnover products are recycled (Goodell 1985; Goodell and Schwarz 1985; Park and Uehara 2008). To prevent cell lysis, PG synthesis needs to be coordinated with PG hydrolysis. In *E. coli*, two multiprotein complexes, the elongasome and the divisome, facilitate cell length growth and cell division, respectively (den Blaauwen et al. 2008). In both the elongasome and the divisome complexes, PG transpeptidase activities and glycosyltransferase activities are essential for the synthesis of nascent PG. RodA and PBP2, integral components of the elongasome complex, provide glycosyltransferase and transpeptidase activities, respectively (Sjodt et al. 2018; Van der Ploeg, Goudelis, and Den Blaauwen 2015). MreC and MreD, two additional members of the elongasome complex, are responsible for mediating the synthesis of nascent PG during cell elongation (Liu et al. 2020; Rohs et al. 2018; Wachi et al. 1989).

Under the ultimate control of the tubulin homologue FtsZ, the divisome promotes the synthesis of septal PG at the division site (Bi and Lutkenhaus 1991). In this complex, PBP1B, FtsW, and PBP3 (FtsI) provide PG synthesis activity (Bertsche et al. 2006; Cho et al. 2016; Leclercq et al. 2017; Müller et al. 2007). FtsW and PBP3 are recruited to the division site by the FtsBLQ subcomplex, which inhibits the glycosyltransferase (GTase) activity of PBP1B and the transpeptidase (TPase) activity of PBP3 that is coupled with the GTase activity of FtsW (Taguchi et al. 2019). The assembly of FtsN in the divisome relieves the inhibitory effect of the FtsBLQ subcomplex on PBP1B and PBP3‐FtsW and promotes septal PG synthesis. FtsN is considered as the “trigger” for septal PG synthesis and cell constriction at the division site, and together with ZipA, links PBP1B to the cytosolic part of the initiating divisome at the beginning of cell division (Lyu et al. 2022; Mueller, Westfall, and Levin 2020; Pazos et al. 2018). The synthesis and hydrolysis of septal PG can further accelerate the recruitment of FtsN via its SPOR domain, which binds to denuded PG glycan strands (Gerding et al. 2009; Ursinus et al. 2004). The denuded PG glycan strands are generated through the action of amidases (AmiA, AmiB, and AmiC), which cleave between the peptide and glycan strand and are the main hydrolases for separation of daughter cells (Heidrich et al. 2001; Peters, Dinh, and Bernhardt 2011).

The division machinery in *E. coli* consists of over 20 proteins, and these proteins assemble at the division site in an interdependent and sequential manner (Aarsman et al. 2005). Based on the timing of division protein assembly, they can be categorized into two distinct groups: “early” proteins and “late” proteins. The “early” proteins in the divisome include FtsZ and its membrane anchors FtsA and ZipA and a small amount of FtsN (Goehring, Gonzalez, and Beckwith 2006; Pazos et al. 2018). The ABC transporter FtsEX is recruited to the divisome site after the “early” proteins and involved in the assembly of other divisome proteins (Du et al. 2019). FtsK is the initial “late” protein recruited to the divisome, followed by the FtsBLQ subcomplex, the PBP3‐FtsW pair, and finally the majority of FtsN (den Blaauwen, Hamoen, and Levin 2017).

PG hydrolysis is essential for the expansion and growth of PG (Singh et al. 2012). In *E. coli*, PG hydrolases are involved in cell growth, daughter cell separation, morphogenesis, and PG maturation. Compared with proteins involved in PG synthesis, the hydrolases show a higher apparent redundancy (van Heijenoort 2011). The PG degradation enzymes can be classified into lytic transglycosylases, amidases, and peptidases. The peptidases can be divided into two subgroups, endopeptidases and carboxypeptidases, with endopeptidases cleaving crosslinks between cross‐linked peptide chains and carboxypeptidases removing C‐terminal D‐alanine from tetra‐ or pentapeptides (Matsuhashi et al. 1979; Voedts et al. 2021; Vollmer, Blanot, and de Pedro 2008). Amidases facilitate the separation of daughter cells by removing peptides from glycan chains (Priyadarshini, de Pedro, and Young 2007).

*Escherichia coli* has eight endopeptidase paralogues, MepA, MepM, MepH, MepS, MepK, AmpH, PBP4, and PBP7 (Voedts et al. 2021). Due to the net‐like structure of PG, the “space‐maker” function of hydrolases is hypothesized to be essential for the insertion of nascent PG units (Burman and Park 1984; Höltje 1998). MepM, MepH, and MepS were shown to be redundantly essential for PG expansion during cell elongation (Singh et al. 2012). The absence of MepS significantly increases the cell diameter, and a Δ*mepS* Δ*mepM* mutant loses viability in rich medium (Jeon and Cho 2022). The overexpression of MepH and PBP7 can alleviate the EDTA‐sensitive phenotype of Δ*mepS* (Park et al. 2020). MepK exhibits LD‐endopeptidase activity specific for 3–3 cross‐links (Chodisetti and Reddy 2019). PBP4, encoded by *dacB*, has DD‐endo‐ and carboxypeptidase activity (Korat, Mottl, and Keck 1991) and assists amidases in promoting daughter cell separation (Verheul et al. 2022). The roles of other endopeptidases in PG metabolism are currently not well understood. Due to the redundancy and interchangeability of endopeptidases, their functions have primarily been elucidated through the introduction of multiple genomic deletions and the phenotypes of the resulting mutants (Heidrich et al. 2002). Therefore, identifying the role of the individual endopeptidases is challenging.

In this work, we explored the function of PBP7 (encoded by *pbpG*), using specific antibodies against PBP7 to determine its cellular localization characteristics. We show that active site residues (S67, S124 and K231) are essential for substrate binding and the C‐terminal α helix is essential for its midcell localization but not for substrate binding. Interestingly, the midcell localization of PBP7 relies on an active divisome. We found that the absence of PBP7 influences the timing of midcell assembly of the divisome proteins FtsZ and FtsN. The timing of FtsZ and FtsN localization is further aggravated in the absence of both PBP7 and PBP4. PBP7 especially extends the assembly period of FtsN in the divisome, which might be caused by a reduced septal PG synthesis rate in the Δ*pbpG* mutant.

## 2 Results

### 2.1 PBP7 Localizes Both at the Lateral Wall and Midcell

Knowing the subcellular localization of proteins is essential for dissecting their function. In this study, purified antibodies against PBP7 were used for immunolabeling experiments on wild‐type *E. coli* LMC500 cells cultivated to steady state in GB1 medium at 28°C. Western blot showed that the primary PBP7 antibody contained some antibodies that can bind to other proteins in *E. coli* (Figure S1A). Antiserum was therefore incubated with the Δ*pbpG* mutant to adsorb antibodies that did not specifically bind PBP7, and the remaining antibodies recognizing PBP7 present in the supernatant (Figure S1B) were used for immunolabeling PBP7 in strains.

**FIGURE 1:**
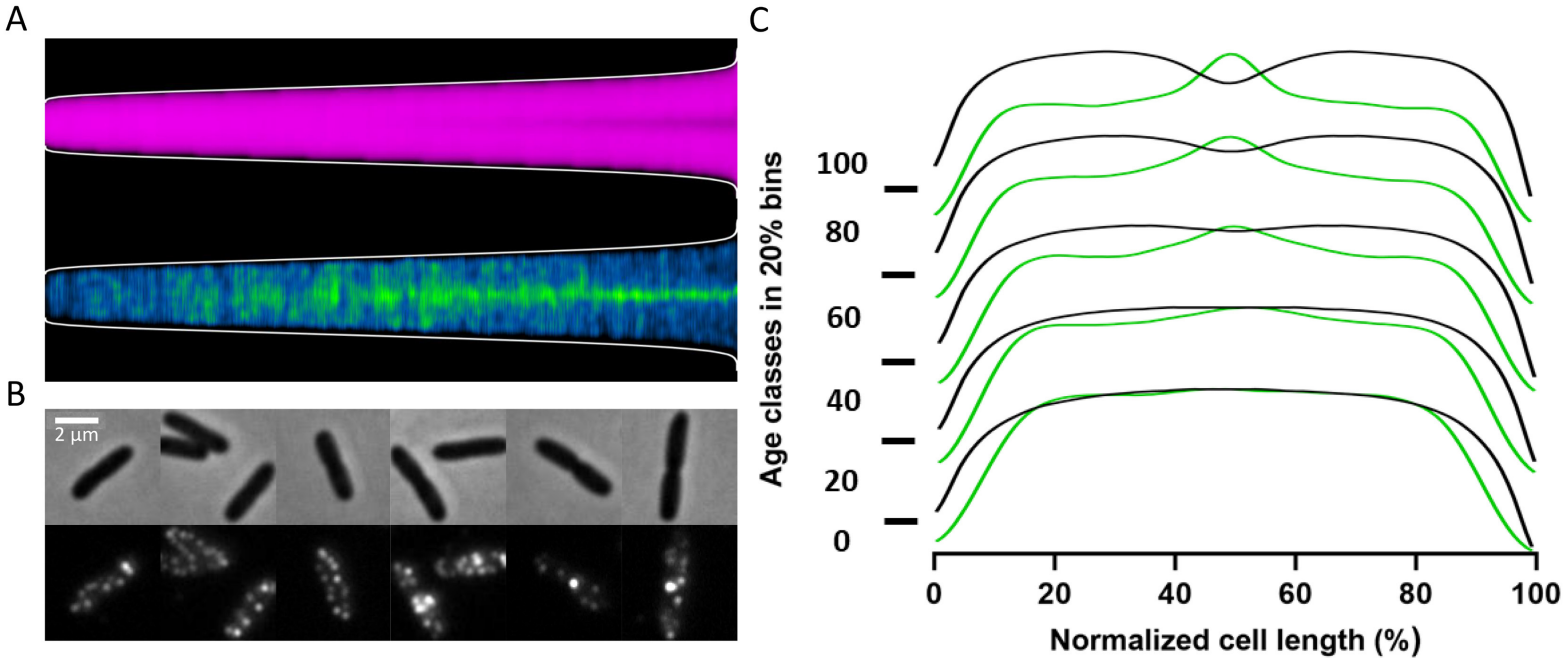
Localization of PBP7 in wild‐type cells. Strain LMC500 was grown to steady state in GB1 medium at 28°C, fixed by 2.8% formaldehyde and 0.04% glutaraldehyde (FA/GA), and subjected to immunolabeling using purified antibodies for PBP7. (A) Diameter (magenta) and PBP7 fluorescence (cyan/green) were plotted according to their cell length (ascending from left to right). (B) Images of the immunolabeled cells. The upper panel shows phase‐contrast images of representative cells, and the lower panel their corresponding fluorescence images. The scale bar equals 2 μm (C) The cell diameter (black lines) and fluorescence (green lines) profiles along the normalized cell length were plotted in 20% age class bins. More than 10,000 cells were analyzed.

When the localization of PBP7 was observed in a small number of cells, it did not exhibit clear localization characteristics (Figure S1C). However, by analyzing the localization of PBP7 in thousands of cells, PBP7 was found to localize in the lateral wall and at the division site of deeply constricting cells (Figure 1A–C). PBP7 was fused with mCherry at its N terminus in its genomic locus. The resulting DsbA^ss^‐mCherry‐PBP7 protein was expressed under the IPTG‐inducible p*trc* promoter, and it exhibited similar localization characteristics to the wild‐type PBP7 in live cells (Figure S1D,E). The cleavable DsbA signal sequence (DsbA^ss^) allows translocation of the fused mCherry‐PBP7 to the periplasm. Possibly due to the instability of mCherry‐PBP7 (Figure S1A), its midcell localization appeared weaker compared to the wild‐type PBP7 in fixed cells (Figure 1C, Figure S1E).

To examine whether the stronger PBP7 signal at midcell was caused by the double membrane overlap of two daughter cells during cell division, we stained the cell membranes with bodipy. The PBP7 signal at midcell was much stronger than the bodipy signal (Figure 1A–C, Figure S2A–C), showing that PBP7 is enriched at the division septum.

### 2.2 Amino Acid Residues S67, S124, and K231 Are Essential for PBP7 Activity

**FIGURE 2:**
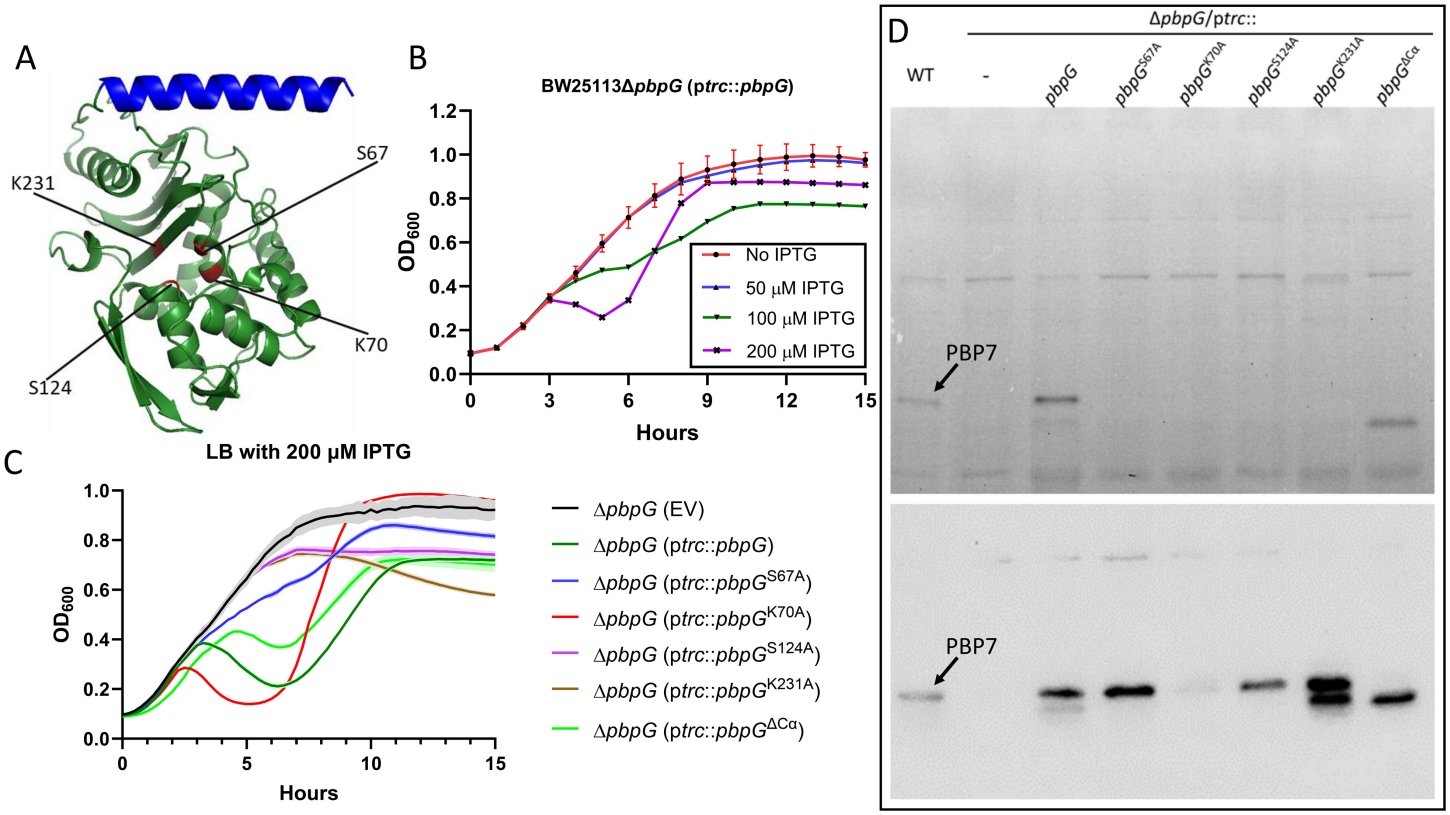
Essential amino acids for PBP7 activity. (A) The structure of PBP7 has been predicted using AlphaFold2 and labeled with PyMOL. The potential active site residues (S67, K70, S124, and K231) are labeled in red color, while the C‐terminal α‐helix (A287‐D312) is labeled in blue color. (B) The toxicity of PBP7 was assessed by monitoring the growth of Δ*pbpG* cells in LB medium at 37°C, with the expression of the *pbpG* gene induced by varying concentrations of IPTG (0, 50, 100, or 200 μM). Growth curves were performed in triplicate for each strain. Time in hours is plotted against the OD measured at 600 nm. The error bars represent their standard deviation (SD). (C) The toxicity of wild‐type PBP7, its four variants, and truncated PBP7 were assessed by monitoring the growth of Δ*pbpG* cells in LB medium at 37°C, with the expression of the *pbpG* gene induced by 200 μM IPTG. The Δ*pbpG* strain with the empty vector (EV) served as a control. The growth curves were performed in triplicate for each strain. Time in hours is plotted against the OD measured at 600 nm. The solid lines and their corresponding shaded areas represent the mean ± SD. (D) The fluorogram in the upper panel shows the binding of Bocillin‐FL to wild‐type PBP7, its four variants, and truncated PBP7, all without IPTG‐induced expression in the Δ*pbpG* mutant. The lower panel shows the expression level of these proteins by immunoblot analysis using the PBP7 antibody.

According to the UniProt database, four amino acids have been predicted to be important for the function of PBP7. S67 and K70 belong to the SXXK motif, which is conserved in PBPs. The S67 in the SXXK motif is the nucleophile residue that attacks the penultimate D‐alanine residue and forms an acyl‐enzyme intermediate with the peptide chain (Macheboeuf et al. 2006; Nicola et al. 2005). The K70 in the SXXK motif might serve as a proton acceptor for the nucleophile attack and assist in forming the intermediate (Zhang et al. 2007). In contrast to the high molecular mass PBP intermediate, the deacylation process of the low molecular mass PBP intermediate involves the use of a water molecule as an acceptor, rather than an adjacent peptide chain (Nicola et al. 2005; Zhang et al. 2007). S124 and K231 belong to the conserved SXN motif and KTG motif, respectively. The S124 and K231 might form a hydrogen bridge with a water molecule to assist the deacylation process of the intermediate (Nicola et al. 2005; Zhang et al. 2007). In addition, the K231 of the KTG motif might serve as an electrostatic anchor for the substrate binding (Zhang et al. 2007). S67, K70, S124, and K231 are thus potentially active residues of PBP7. The structure of PBP7, simulated using AlphaFold (Jumper et al. 2021), revealed that the C‐terminal α‐helix containing amino acid residues A287‐D312 is distanced from the main structure of PBP7, suggesting that it may be able to interact with other proteins or the cell membrane (Figure 2A).

To validate this prediction, these four active site residues were replaced by alanine using site‐directed mutagenesis on a plasmid‐expressing the *pbpG* gene, creating four variants of PBP7. In addition, the variant lacking the C‐terminal α‐helix (PBP7^ΔCα^) and wild‐type PBP7 were expressed from plasmid using the IPTG‐inducible p*trc* promoter. The overproduction of PBP7 was found to be toxic and resulted in cell lysis, presumably because of spurious PG hydrolase activity (Figure 2B). The overproduction of three variants (S67A, S124A, and K231A) in cells did not exhibit strong toxicity as was observed upon the overproduction of wild‐type PBP7 or the PBP7 variant K70A (Figure 2C). PBP7^ΔCα^ was also toxic, indicating that the C‐terminal α‐helix is probably not essential for the enzymatic activity of PBP7 (Figure 2C). In addition, the regrowth of cells expressing wild‐type PBP7, PBP7 variant K70A, or PBP7^ΔCα^ after cell lysis may be due to the emergence of suppressor mutations or a small portion of bacteria containing lower copy number plasmids, which survived and became dominant.

To test whether the four PBP7 variants and truncated PBP7 were able to bind a substrate, a Bocillin FL‐binding assay was carried out. Bocillin FL, a fluorescent derivative of penicillin, binds to the active site of PBPs (Zhao et al. 1999). As shown in Figure 2D‐upper half, the three variants (S67A, S124A, and K231A) of PBP7 did not bind Bocillin FL, while the truncated PBP7 retained its ability to bind Bocillin FL. These three PBP7 variants (S67A, S124A, and K231A) were less toxic (Figure 2C) and unable to bind Bocillin‐FL (Figure 2D), indicating that these three amino acids are critical to its function. The PBP variants were expressed at higher levels than the endogenous protein in wild‐type cells, except for K70A which was very toxic and presumably killed the cells before a higher amount accumulated (Figure 2D‐lower panel).

### 2.3 The C‐Terminal α‐Helix but Not Activity Is Needed for Midcell Localization

**FIGURE 3:**
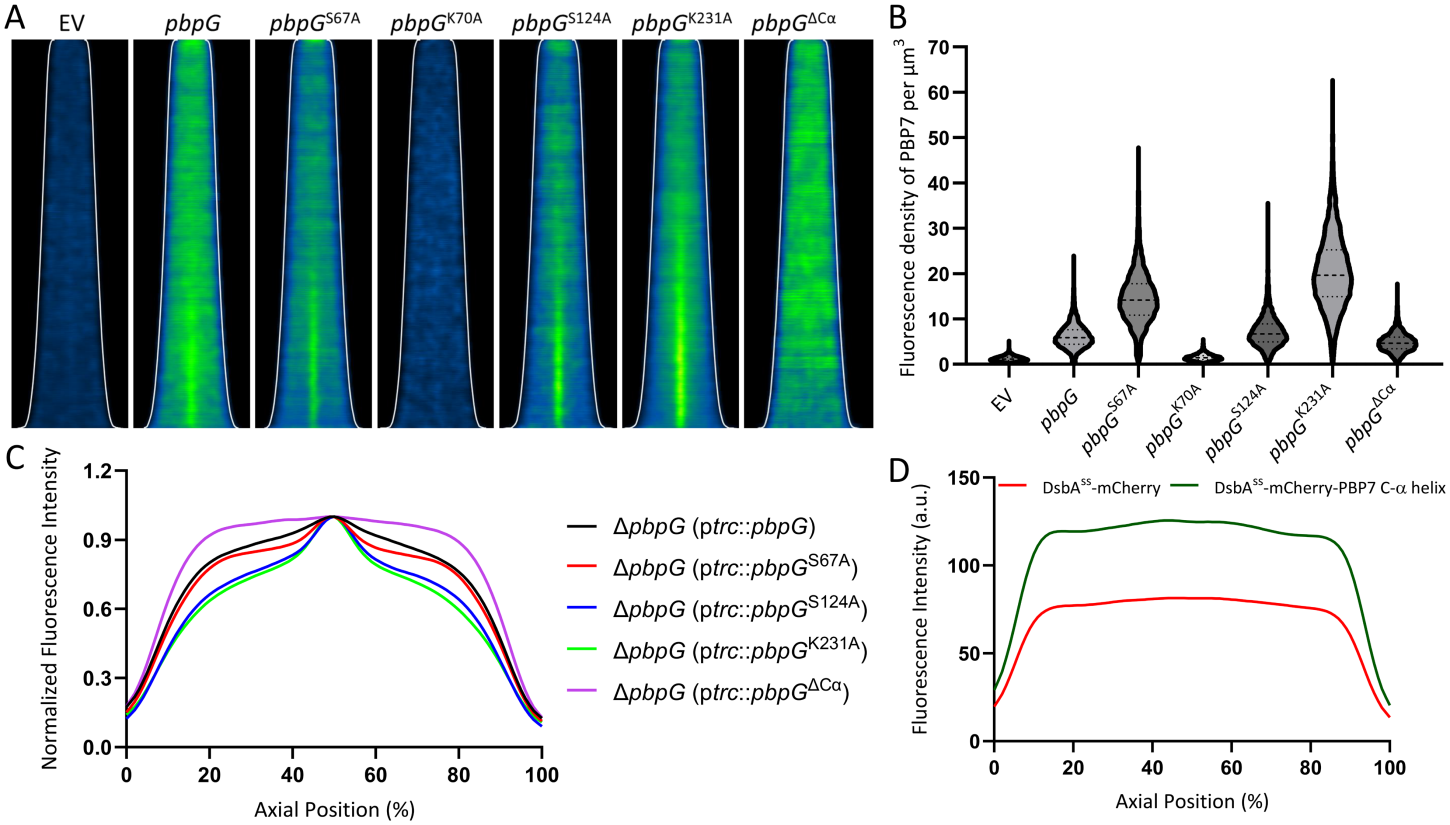
PBP7 midcell localization is independent of its activity and dependent on its C‐terminal α‐helix. (A) The Δ*pbpG* strain was transformed with the parental empty vector (EV) or vectors expressing wild‐type PBP7, its four different variants, or the C‐terminal α‐helix truncated PBP7. Cells were grown to steady state in GB4 medium at 28°C, fixed by FA/GA, and subjected to immunolabeling using purified antibodies for PBP7. The brightness and contrast of the demographs were adjusted to enhance the visibility of PBP7 localization and do therefore not reflect the amount of PBP7 in samples. The number of cells analyzed was more than 3000 cells for each sample. (B) The fluorescence density (intensity per μm^3^) was used as a proxy for evaluating the relative concentration of PBP7 in the different strains, labeled as in panel A. (C) The average normalized fluorescence intensity of cells expressing wild‐type PBP7, three PBP7 variants (S67A, S124A, and K231A), or the C‐terminal α‐helix truncated PBP7 was plotted along the cell's axial position. (D) The cells harboring a plasmid expressing DsbA^ss^‐mCherry or DsbA^ss^‐mCherry‐C‐terminal α‐helix of PBP7 were cultured in GB4 medium to steady state and fixed by FA/GA. The average fluorescence intensity in cells obtained from the demographs was plotted along their axial position in arbitrary units (a.u.).

To investigate the dependence of PBP7 midcell localization on its activity and the presence of its C‐terminal α‐helix, the wild‐type PBP7, four variants, and PBP7^ΔCα^ were expressed in Δ*pbpG* grown in minimal glucose medium at 28°C without induction to avoid toxicity. Their localization was assessed using the purified antibody. The demographs sort the cells based on their length. Each cell is displayed as a horizontal one‐pixel line that contains all fluorescence intensity of the width of the cell at that pixel. For unknown reason, the three inactive PBP7 variants (S67A, S124A, and K231A) exhibited somewhat stronger midcell localization compared to the wild‐type PBP7 (Figure 3A–C, Figure S3). However, the variant lacking the C‐terminal α‐helix did not localize at midcell. To verify whether the α‐helix was the determining factor for midcell localization, DsbA^ss^‐mCherry or the DsbA^ss^‐mCherry‐C‐terminal α‐helix of PBP7 (A287‐D312) fusion was expressed in wild‐type cells (BW25113) cultured in GB4 medium at 28°C to steady state and fixed by a formaldehyde/glutaraldehyde mixture (FA/GA). No significant midcell localization was observed for the mCherry constructs indicating that the α‐helix of PBP7 is required but not sufficient for PBP7 localization at midcell (Figure 3D). Alternatively, mCherry changed the affinities of the C‐terminal α‐helix of PBP7 for its partners.

### 2.4 PBP7 Midcell Localization Depends on the Presence of FtsZ and FtsN, and Activity of PBP3

The midcell localization of PBP7 suggests its potential involvement in the divisome. To investigate PBP7's relationship with cell division proteins, cells lacking different essential proteins in the divisome were cultured in GB4 medium at 28°C. The cells were immunolabeled with purified antibodies for PBP7.

**FIGURE 4:**
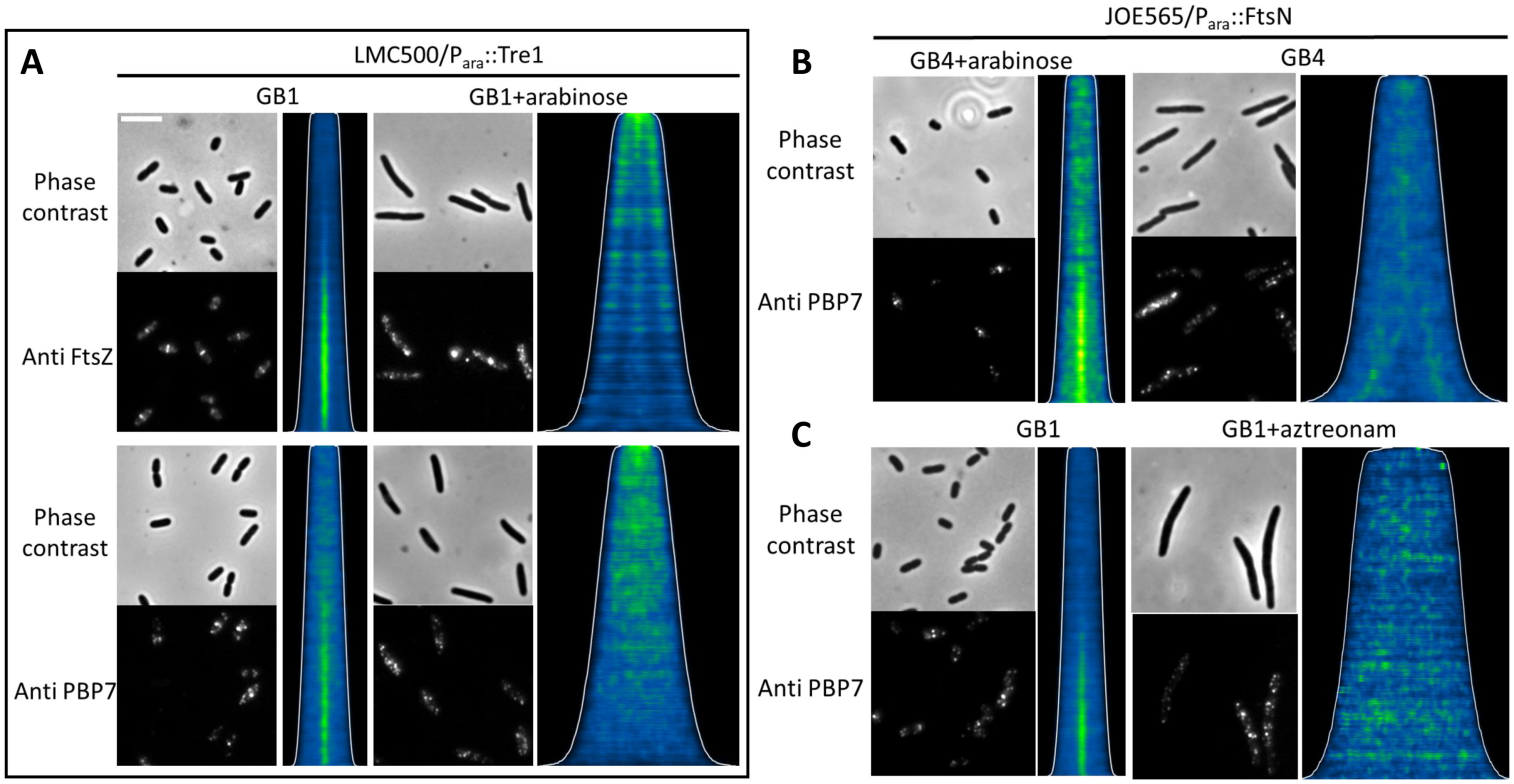
The midcell localization of PBP7 is dependent on an active divisome. (A) Cells were grown to steady state in GB1 medium at 28°C and the expression of Tre‐1 was either not induced or induced with 0.2% arabinose for another two mass doubling times. The cells were fixed by FA/GA and immunolabeled with antibodies against FtsZ and PBP7. The number of cells analyzed was more than 1500 cells for each sample. (B) JOE565 *ftsN*::*kan* ^*R*^/pBAD‐FtsN cells grown to steady state in GB4 medium with 0.2% arabinose at 28°C were washed with pre‐warmed GB4 medium to remove arabinose in medium. Subsequently, the cells were cultivated in GB4 medium with or without 0.2% arabinose for another two mass doubling times. The number of cells analyzed were 4058 and 2011 for with and without arabinose inducing, respectively. (C) Wild‐type strain LMC500 was grown to steady state in GB1 medium at 28°C and continued to grow in GB1 medium without or with 1 μg mL^−1^ aztreonam for another two mass doubling times at 28°C. The number of cells analyzed was 1600 and 6131 for with and without aztreonam inhibition, respectively. For all panels, cells were fixed by FA/GA and immunolabeled with antibodies against PBP7. The phase‐contrast image, corresponding fluorescence image, and demographs of cells sorted according to cell length are present for each sample. The scale bar equals 5 μm.

FtsZ, the essential scaffold for the assembly of the divisome, can undergo modification by Tre1 (type VI secretion ADP‐ribosyltransferase effector 1), resulting in the inhibition of its polymerization capacity (Ting et al. 2018). The localization of FtsZ and PBP7 in cells expressing Tre1 was assessed through immunolabeling using a specific antibody for FtsZ (Aarsman et al. 2005) and purified antibodies for PBP7. As shown in Figure 4A, the expression of Tre1 in cells, which prevents the assembly of FtsZ at the division site, leads to the loss of midcell localization of PBP7.

FtsN was produced from the pBAD‐FtsN plasmid under the control of an arabinose inducible promoter in strain JOE565, which harbors the *ftsN*::*kan* ^*R*^ inactivating insertion on its genome (Chen and Beckwith 2001). In medium without arabinose, FtsN becomes depleted, resulting in filamentous cell growth. As shown in Figure 4B, the depletion of FtsN resulted in the loss of midcell localization of PBP7.

The absence or the inhibition of the divisome TPase PBP3 is known to cause cell filamentation (Pogliano et al. 1997). As expected, we observed filamentous cells after growing cells for two mass doublings in the presence of the PBP3 inhibitor aztreonam (Davies et al. 2007; Sykes et al. 1982). Interestingly, the aztreonam‐treated cells lost the midcell localization of PBP7 (Figure 4C). Because PBP3 was still present in the divisome in the presence of aztreonam (Figure S4A,B), we concluded that active PBP3 was essential for the midcell localization of PBP7, and the inactivated enzyme was not sufficient.

To identify potential proteins that interact with PBP7 and contribute to its midcell localization, we checked the localization of PBP7 in various mutants associated with the divisome by immunofluorescence using the anti‐PBP7 antibody, as well as in mutants lacking proteins reported to be possible partners of PBP7 (Bertsche et al. 2006; Du et al. 2019; Gray et al. 2015; Kang and Boll 2022; Magnet et al. 2008; Peters et al. 2013; Romeis and Höltje 1994; Sauvage et al. 2008; Tsang, Yakhnina, and Bernhardt 2017; Yakhnina and Bernhardt 2020). PBP7 localized at midcell in all analyzed mutants (Figure S5A–C). Interestingly, a defect in the Tol‐Pal system, which is required for completing daughter cell separation, resulted in a stronger midcell localization of PBP7. This might be caused by insufficient hydrolysis of septal PG, which would leave more substrate for PBP7 at midcell.

### 2.5 PBP7 Influences the Timing of FtsN and FtsZ Recruitment to the Divisome

**FIGURE 5:**
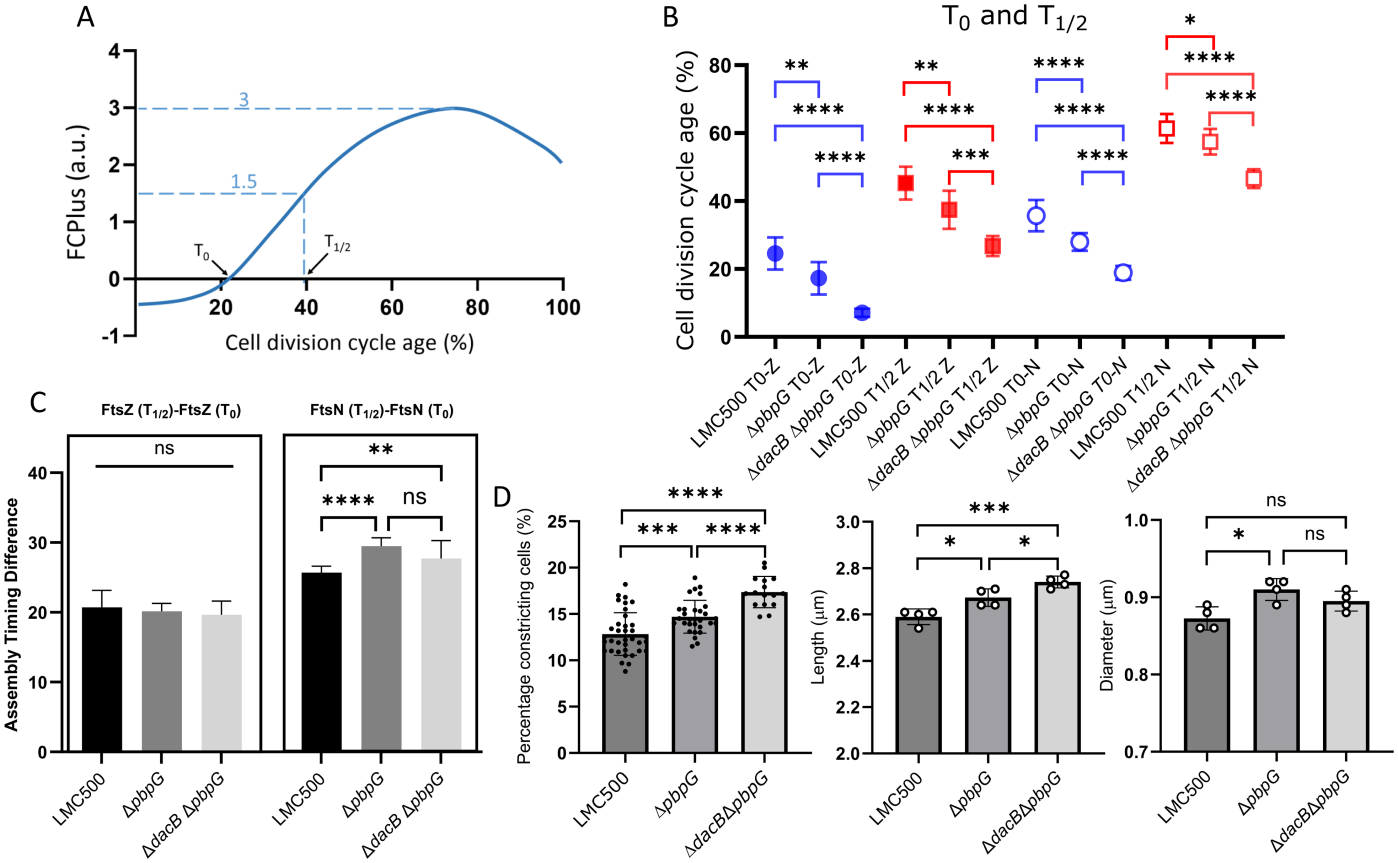
PBP7 affects the timing of divisome assembly. (A) Graphical representation illustrating the meaning of *T*_0_ and *T*_1/2_. (B) Cell division cycle age timing of FtsZ and FtsN for the Δ*pbpG* strain, Δ*dacB* Δ*pbpG*, and its parental LMC500. Each value is obtained from at least 7 replicates, symbols indicate the mean, and error bars represent their SD. (C) The assembly timing difference of FtsZ (*T*_1/2_ − *T*_0_) and FtsN (*T*_1/2_ − *T*_0_) in Δ*pbpG* strain, Δ*dacB* Δ*pbpG*, and its parental LMC500. Each value is obtained from at least seven replicates, bars indicate the mean, and error bars represent their SD. (D) The cell length, cell diameter, and percentage of constricting cells of wild‐type, Δ*pbpG*, and Δ*dacB* Δ*pbpG* strains cultured in GB1 medium at 28°C. The bars indicate the mean, and error bars represent their SD. Each dot represents one replication for the strain. An unpaired *t*‐test with *p* > 0.05 = ns, *p* ≤ 0.05 = *, *p* ≤ 0.01 = **, *p* ≤ 0.001 = ***p ≤ 0.0001 = **** were used for the data in panel (B–D).

To further dissect the impact of PBP7 on the divisome, the assembly timing of the earliest and latest midcell localization proteins FtsZ and FtsN, respectively, was investigated (Verheul et al. 2022). LMC500 and LMC500 Δ*pbpG* were cultured in GB1 medium at 28°C to steady state. The cells were fixed using FA/GA and subsequently labelled with antibodies against FtsZ and FtsN. The division cycle begins when the previous division is completed and it ends when the daughter cells are separated. The division cycle age was normalized from 0 to 100 (Figure 5A). Based on the normalized division cycle age, the assembly timing of divisome proteins was determined by comparing the extra amount of fluorescence at midcell compartment (± 0.2 μm from the cell center) to the rest of the cell (FCPlus). In one cell division cycle, the *T*_0_ refers to the point at which the fluorescence at the midcell is equivalent to the fluorescence in the rest of the cells (Figure 5A). When the fluorescence value at midcell reaches half of its maximum, we refer to that time point as *T*_1/2_ (Figure 5A). The *T*_0_ and *T*_1/2_ for FtsZ and FtsN in Δ*pbpG* were significantly earlier in the cell division cycle compared to those in the wild‐type strain (Figure 5B). A deletion of the PBP4 gene, *dacB*, also caused an earlier divisome assembly (Verheul et al. 2022).

To assess whether deleting the genes of both PBP4 and PBP7 would have an additive effect, an additional mutation of *dacB* was introduced into the Δ*pbpG* strain. The *T*_0_ and *T*_1/2_ for FtsZ and FtsN in Δ*dacB* Δ*pbpG* significantly preceded that of the wild‐type and Δ*pbpG* strains (Figure 5B). This suggests that PBP4 and PBP7 individually influence the timing of FtsN and FtsZ assembly in the divisome. It is possible that the DD‐carboxypeptidase activity of PBP4 has an additional effect on the timing of FtsN and FtsZ arrival to the divisome.

The accumulation period (*T*_0_ to *T*_1/2_) of FtsZ showed no statistical difference among wild‐type, Δ*pbpG*, and Δ*dacB* Δ*pbpG* (20.7%, 20.15%, and 19.64% of the cell division cycle, respectively, Figure 5C). In contrast, the accumulation period of FtsN is significantly shorter in the wild type with 25.7% of the cell division cycle compared to the Δ*pbpG* (29.5%) and Δ*dacB* Δ*pbpG* (27.7%). The accumulation period of FtsN between Δ*pbpG* and Δ*dacB* Δ*pbpG* mutants did not show a statistically significant difference (Figure 5C). These results suggest that the absence of PBP7 slows down the assembly of FtsN at the divisome. The additional deletion of *dacB* in the Δ*pbpG* mutant does not further impede the assembly of FtsN in the divisome.

The Δ*pbpG* mutant cells were longer, wider, and more cells were in the process of constriction compared to wild‐type cells, suggesting that PBP7 might play a role in mediating elongasome activity and daughter cell separation (Figure 5D). Since MepS and MepM are likely the primary endopeptidases for cell elongation, we primarily investigated the role of PBP7 in the divisome. In the Δ*envC* strain, which exhibits a defect in daughter cell separation, we overproduced wild‐type PBP7, inactive PBP7^S67A^, and MepS (another endopeptidase serving as a control), using different IPTG concentrations. Only the overproduction of wild‐type PBP7 reduced the cell length of Δ*envC* mutant with increasing IPTG concentration (Figure S6). This suggests that PBP7 possesses the activity to assist in the daughter cell separation of the Δ*envC* mutant. In addition, the extra mutation of *dacB* in the Δ*pbpG* strain leads to longer cells and a higher percentage of constricting cells compared with the Δ*pbpG* mutant (Figure 5D). Changes in cell length and the percentage of cell constrictions can reflect alterations in cell division. Similar to cells lacking PBP4, the increased cell length and a higher percentage of constricting cells in Δ*pbpG* mutant compared with its parental strain might be caused by delayed daughter cell separation. FtsZ and FtsN disassemble from the old divisome and relocate to the future division site before cell division finishes and the daughter cells separate. The relatively slower cell separation in the Δ*pbpG* mutant might lead to the earlier localization of FtsZ and FtsN at the division site of daughter cells. The further increase in cell length and cell constriction ratio in the Δ*dacB* Δ*pbpG* mutant compared with the Δ*pbpG* mutant aligns with our observations regarding the *T*_0_ and *T*_1/2_ values for FtsZ and FtsN in the Δ*dacB* Δ*pbpG* strain, which significantly precede those in the wild‐type and Δ*pbpG* strains (Figure 5D).

### 2.6 PBP7 Influences Septal PG Synthesis

**FIGURE 6:**
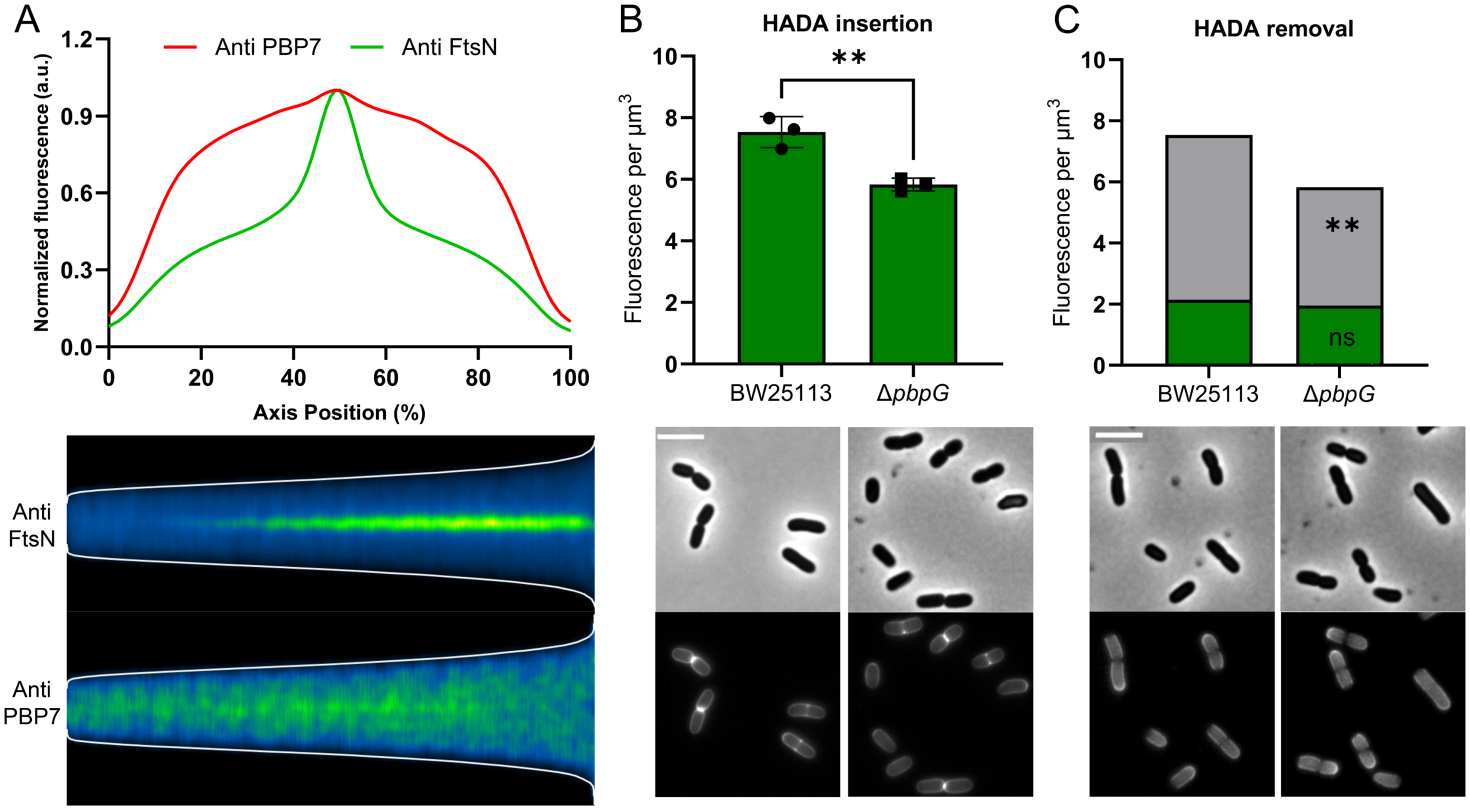
The localization of PBP7 and FtsN in PBP3ts strain and the influence of PBP7 on PG synthesis. (A) PBP3ts cells grown to steady state in GB4 medium at 28°C were fixed with FA/GA and subjected to immunolabeling using antibodies against PBP7 and FtsN. The fluorescence density of FtsN and PBP7 was plotted along the cell's axial position. The fluorescent signal was normalized against the maximal signal density. (B) Fluorescence per μm^3^ of incorporated HADA in wild‐type BW25113 and in Δ*pbpG* (dots indicate fluorescence per μm^3^ for three individual replicates). Unpaired *t*‐test: *p* ≤ 0.01 = **. Bottom: phase‐contrast and corresponding fluorescence images of HADA‐labelled cells of BW25113 and Δ*pbpG*. The scale bar equals 5 μm. (C) Fluorescence per μm^3^ of removed HADA (gray color bar) and remaining HADA (green color bar) in wild‐type BW25113 and in Δ*pbpG* (dots indicate fluorescence per μm^3^ for three individual replicates) after growth for 20 min in the absence of HADA. Each value is obtained from three individual replicates. Unpaired *t*‐test, *p* > 0.05 = ns, *p* ≤ 0.01 = **. Bottom: phase‐contrast and corresponding fluorescence images of HADA‐depleted cells of BW25113 and Δ*pbpG*. The scale bar equals 5 μm.

FtsN assembly is influenced by interaction with divisome proteins, new septal PG synthesis, and the accumulation of denudated PG in the division site. The connection between PBP7 and FtsN was examined in a PBP3ts strain, which harbors the temperature‐sensitive PBP3^G191D^ allele. We observed that PBP7 lost its midcell localization already at the permissive temperature in PBP3ts cells, whereas FtsN was still localized at the cell division site (Figure 6A). This result suggests that FtsN and PBP7 might not directly interact physically in PBP3ts cells. Additionally, this PBP3^G191D^ protein seemed to be unstable even at 28°C as the cells were longer and had lower PBP3 signal intensity than the parental strain LMC500 (Figure S7A–C).

To determine the potential influence on PG synthesis by PBP7, a HADA incorporation assay was carried out. The fluorescent D‐alanine derivative 7‐hydroxycoumarincarbonylamino‐D‐alanine (HADA) is incorporated into PG by transpeptidases, which makes HADA a suitable tool to study the cellular activity of these enzymes and a reasonable proxy for PG synthesis. The HADA incorporation into PG was less in the Δ*pbpG* strain compared to in the wild type (Figure 6B). This could explain the extended assembly period (*T*_0_ to *T*_1/2_) of FtsN in the divisome of the Δ*pbpG* strain compared to wild‐type cells. The removal of HADA from wild‐type and Δ*pbpG* cells was also assessed after the cells were washed twice and cultured for an additional 20 min. The remaining HADA was similar between the two strains and more HADA was removed in wild‐type cells compared with Δ*pbpG* cells, suggesting that wild‐type cells might have stronger PG degradation than Δ*pbpG* cells (Figure 6C). Similar to MepS and MepM, which create space for newly synthesized PG insertion in the lateral wall, PBP7 might act as a “space‐maker” in the divisome. The absence of PBP7 might delay the insertion of nascent septal PG at the division site.

### 2.7 PBP7 Does Not Influence the Amount of Septal Denuded Glycan Chains

The generation of denuded glycan chains at the septal PG can be visualized by the protein fusion mCherry‐SPOR^FtsN^. The SPOR domain, which is found in many bacteria, has been shown to bind denuded PG (Gerding et al. 2009). *Escherichia coli* encodes four proteins (DamX, DedD, FtsN, and RlpA) that contain this domain (Arends et al. 2010; Gerding et al. 2009). In our work, the SPOR domain from FtsN was fused to DsbA^ss^‐mCherry under the control of the p*trc* promoter. The fusion protein is transported into the periplasm by the cleavable DsbA signal sequence (DsbA^ss^). The fused protein was expressed in wild‐type, Δ*pbpG*, and Δ*amiC* strains. AmiC is an amidase in *E. coli*, the Δ*amiC* mutant was used as a negative control to assess the amount of septal denuded glycan chains.

**FIGURE 7:**
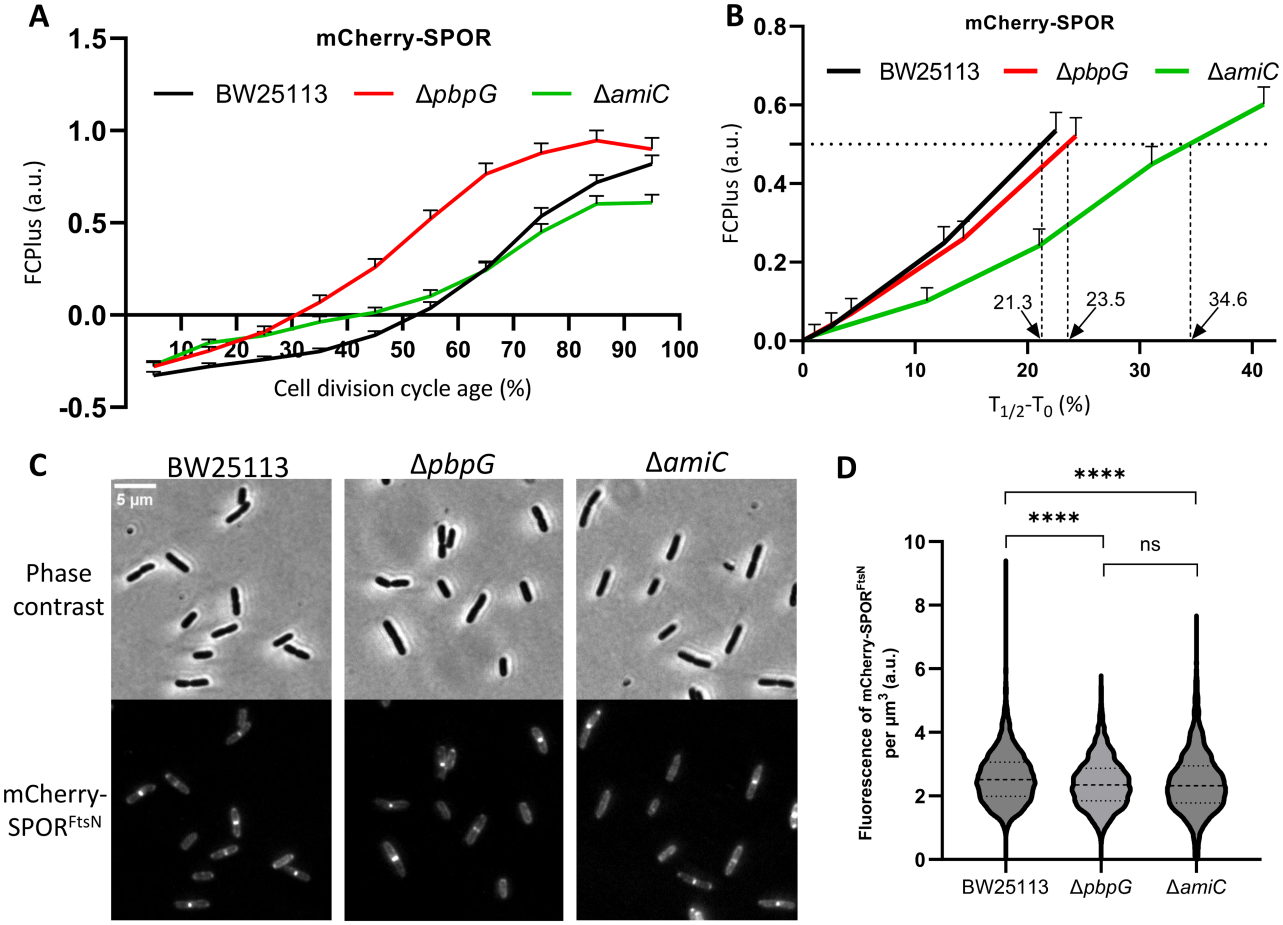
The timing of localization of mCherry‐SPOR^FtsN^ at the division site. (A) The timing of the localization of mCherry‐SPOR^FtsN^ at midcell in the wild‐type BW25113, Δ*pbpG*, and Δ*amiC* strains during the cell division cycle age (%). The number of cells analyzed was 2892, 1858, and 2250 for BW25113, *ΔpbpG*, and *ΔamiC*, respectively. The average FCPlus was measured in 10% age bins and marked with 95% confidence bars. (B) The FCPlus value obtained from a mCherry‐SPOR^FtsN^ fusion was used as a proxy to estimate the relative amount of septal denuded glycan chains in wild‐type, Δ*pbpG*, and Δ*amiC* cells. (C) Phase‐contrast and corresponding fluorescence images of cells of BW25113, Δ*pbpG*. and Δ*amiC*, all of which were harboring a plasmid expressing DsbA^ss^‐mCherry‐SPOR^FtsN^. The scale bar equals 5 μm. (D) The fluorescence concentration of mCherry‐SPOR^FtsN^ in wild‐type, Δ*pbpG*, and Δ*amiC* cells. Unpaired *t*‐test: *p* > 0.05 = ns, *p* ≤ 0.001 = ****.

The signal for denuded chains at the septum was followed by comparing FCPlus over the cell cycle in wild‐type, Δ*pbpG*, and Δ*amiC* strains, all of which were harboring a plasmid expressing DsbA^ss^‐mCherry‐SPOR^FtsN^. The *T*_0_ of DsbA^ss^‐mCherry‐SPOR^FtsN^ in Δ*pbpG* was earlier compared with that in wild‐type cells (Figure 7A). This result is consistent with the earlier FtsZ and FtsN localization timing in divisome assembly of the *ΔpbpG* mutant. To better quantify the formation of denuded chains, we normalized the *T*_0_ of the DsbA^ss^‐mCherry‐SPOR^FtsN^ in the three strains to 0 and compared the cell cycle fraction required to reach an FCPlus value of 0.5, as a proxy for the formation of denuded glycan (i.e., *T*_1/2_). The *T*_1/2_ − *T*_0_ from 0 to 0.5 was 21.3%, 23.5%, and 34.6% cell division cycle time for wild‐type, Δ*pbpG*, and Δ*amiC* cells, respectively (Figure 7B). Compared to wild‐type cells, the denuded glycan chain formed slower in the Δ*amiC* mutant, while there was little difference in the Δ*pbpG* mutant. The DsbA^ss^‐mCherry‐SPOR^FtsN^ in BW25113, Δ*pbpG*, and Δ*amiC* cells shows midcell localization (Figure 7C). Although the fluorescence concentration of DsbA^ss^‐mCherry‐SPOR^FtsN^ in wild‐type strain was higher than that in the Δ*pbpG* and Δ*amiC* strains, their concentrations were close enough (Figure 7D).

To further verify the relationships between amidases, their activators and PBP7, multiple deletion strains were constructed, and the cell phenotypes of strains were analyzed (Table S1). The cell length of single mutants (Δ*amiA*, Δ*amiB*, Δ*amiC*, Δ*envC*, and Δ*nlpD*) did not show a clear difference compared to the respective double mutants (Δ*amiA* Δ*pbpG*, Δ*amiB* Δ*pbpG*, Δ*amiC* Δ*pbpG*, Δ*envC* Δ*pbpG*, and Δ*nlpD* Δ*pbpG*). This implies that PBP7 likely does not have a direct interaction with amidases and their activators.

## 3 Discussion

*E. coli* encodes eight endopeptidase paralogs in its genome. Due to the redundancy and interchangeability of endopeptidases, the study of the specific role of a single endopeptidase becomes challenging. Compared to other members of endopeptidases in *E. coli*, our understanding of the function of PBP7 has been limited. In this study, we investigated the function, localization, and regulation of PBP7.

PBP7 localized at the lateral wall and midcell as shown by immunolabeling with purified antibodies against PBP7 (Figure 1). Through the study of four variants of PBP7, we discovered that the predicted active site amino acids (S67, S124, and K231) are important for the activity of PBP7, but they are not crucial for its midcell localization (Figures 2 and 3). The C‐terminal α‐helix (A287‐D312) of PBP7 is essential for midcell localization without affecting the β‐lactam binding activity of PBP7 (Figures 2D and 3A,C). The mutation K70A enhanced the toxicity of PBP7 compared to wild‐type PBP7 (Figure 2C), which may be counterintuitive and explained by adverse effects of this particular inactive version (but not the others) competing with active PBP7 for binding to other proteins or the substrate. Unexpectedly, the S124A version, which should be impaired in the deacylation process of the acyl‐enzyme intermediate with the peptide chain, did not bind Bocillin FL for unknown reason.

The midcell localization of PBP7 led us to investigate whether PBP7 is part of the divisome. By disrupting the assembly of FtsZ and FtsN at midcell and inhibiting the PG synthesis activity of the divisome using aztreonam, we demonstrated that PBP7 localization depends on the assembly of FtsZ and FtsN in the divisome and on an active divisome (Figure 4). To further investigate the influence of PBP7 on the assembly of the divisome, we examined the midcell arrival timing of FtsZ and FtsN in both wild‐type cells and the Δ*pbpG* mutant. FtsZ is the first and FtsN is the last essential protein to assemble in the divisome. Both proteins assembled in the Δ*pbpG and* Δ*dacB* Δ*pbpG* strains significantly earlier at midcell compared to their parental strain (Figure 5B). This might be caused by a later separation between daughter cells of Δ*pbpG and* Δ*dacB* Δ*pbpG* strains. At some stage during the cell cycle, FtsZ and FtsN are released from the division site of the two daughter cells and move toward the future division site. In a delayed separation process length growth continuous, FtsZ disassembles at the old division site and relocates to the future division site before the separation process finishes, which leads to an early assembly time of FtsZ in the daughter cell. The increased cell length and a higher percentage of constricting cells observed in Δ*pbpG* compared to the wild‐type strain can indirectly prove the possibility of a delayed separation occurring in the Δ*pbpG* strain (Figure 5D). Overproduction of PBP7 in the Δ*envC* strain facilitated the separation of the two daughter cells, indicating that PBP7 possesses the activity to assist in the separation of daughter cells (Figure S4). Another DD‐endopeptidase, PBP4 has previously been shown to support the function of amidases and mediate cell length in the cells lacking amidases or their activators (Priyadarshini, Popham, and Young 2006; Verheul et al. 2022). The longer cell length and higher percentage of constricting cells in Δ*dacB* Δ*pbpG* is also consistent with our speculation that the absence of PBP7 and PBP4 causes a daughter cell separation defect. That might explain the significantly earlier *T*_0_ and *T*_1/2_ of FtsZ and FtsN in the *ΔdacB* Δ*pbpG* mutant compared with wild‐type and Δ*pbpG* cells.

To further assess which process was influenced by PBP7 in cells, we determined the assembly period from *T*_0_ to *T*_1/2_ of FtsZ and FtsN. The FtsZ assembly period from *T*_0_ to *T*_1/2_ did not show a difference among wild‐type, Δ*pbpG*, and Δ*dacB* Δ*pbpG* strains (Figure 5C). However, an increased assembly period from *T*_0_ to *T*_1/2_ of FtsN was observed in the *ΔpbpG* and *ΔdacB* Δ*pbpG* strains compared with wild‐type cells (Figure 5C). The essential proteins in the divisome assemble in a hierarchical manner (Aarsman et al. 2005; Egan and Vollmer 2013). The comparison of “FtsZ (*T*_1/2_) − FtsZ (*T*_0_)” among wild‐type Δ*pbpG* and Δ*dacB* Δ*pbpG* strains did not show any significant differences, implying that PBP7 and PBP4 do not influence the assembly period from *T*_0_ to *T*_1/2_ of FtsZ (Figure 5D). The longer assembly period of “FtsN (*T*_1/2_) − FtsN (*T*_0_)” in the Δ*pbpG* and Δ*dacB* Δ*pbpG* mutants suggests that PBP7 affects the FtsN assembly in the divisome (Figure 5D). FtsN is the trigger of cell constriction at the division site (Lyu et al. 2022; Mueller, Westfall, and Levin 2020). The higher percentage of cells undergoing constriction in the Δ*pbpG* mutant compared to the wild‐type cells also supports the observation that FtsN assembles slower at the divisome site (Figure 5B,D). The assembly period of FtsN (*T*_1/2_ to *T*_0_) in the Δ*pbpG* mutant did not extend further in the Δ*dacB* Δ*pbpG* strain, which indicates that PBP4 has a minor if any effect on FtsN assembly in the divisome (Figure 5D).

To investigate the mechanism of PBP7 affecting the assembly of FtsN, we examined the interplay between FtsN and PBP7, as well as PG synthesis and formation of denuded septal PG glycan chains in both wild‐type and Δ*pbpG* mutant cells. FtsN localized at midcell in the PBP3ts strain, but PBP7 lost its midcell localization at the permissive temperature (Figure 6A), implying that FtsN is not sufficient to recruit PBP7 to preseptal sites. Although unlikely, we cannot strictly exclude that G191 of PBP3 may facilitate an interaction between FtsN and PBP7.

In the divisome, the assembly of FtsN is also influenced by the synthesis of septal PG and formation of denuded glycan chains (Lyu et al. 2022). The activity of transpeptidases was evaluated using a HADA incorporation assay, as a proxy for PG synthesis activity (Mamou et al. 2022; Navarro et al. 2022). A lower concentration of fluorescence HADA was observed in the Δ*pbpG* mutant compared to that in wild‐type cells, suggesting a slower PG synthesis in the Δ*pbpG* mutant and/or faster degradation of the HADA‐labelled PG (Figure 6B). By assessing the remaining HADA in wild‐type and Δ*pbpG* mutant cells after an additional 20 min of culturing, the amount of remaining HADA‐labelled PG in the Δ*pbpG* mutant was comparable with wild‐type cells (Figure 6C). The PG hydrolytic activity in the Δ*pbpG* mutant might be lower than in the wild‐type strain, as suggested by the reduced removal of HADA in the Δ*pbpG* mutant (Figure 6C). PBP7 might act as a “space‐maker” for inserting nascent septal PG in the division site. The absence of PBP7 could slow down the synthesis of septal PG, thereby reducing the efficiency of the divisome and affecting the accumulation period of FtsN in the divisome. This hypothesis is consistent with the suggestion that septal PG degradation activates PG synthesis (Navarro et al. 2022). The influence of PBP7 on the amount of denuded glycan chain material and the rate of its formation was investigated by a fused DsbA^ss^‐mCherry‐SPOR^FtsN^ protein. The SPOR domain can bind the denudated PG (Alcorlo et al. 2019; Gerding et al. 2009). By comparing the cell division cycle time from *T*_0_ to *T*_1/2_ of DsbA^ss^‐mCherry‐SPOR^FtsN^ in wild‐type, Δ*pbpG*, and Δ*amiC*, we found that the absence of PBP7 does not clearly influence the formation of denuded glycan chains (Figure 7B). The minor timing difference (*T*_1/2_ − *T*_0_ of DsbA^ss^‐mCherry‐SPOR^FtsN^) between wild‐type cells and Δ*pbpG* mutant might be caused by slower septal PG synthesis in the Δ*pbpG* mutant. To further test the relationship between amidases and PBP7, single deletions of amidases and amidase activators were introduced in the Δ*pbpG* mutant. The cell length of single mutations (Δ*amiA*, Δ*amiB*, Δ*amiC*, Δ*envC*, and Δ*nlpD*) were not different compared to their respective double mutations (Δ*amiA* Δ*pbpG*, Δ*amiB* Δ*pbpG*, Δ*amiC* Δ*pbpG*, Δ*envC* Δ*pbpG*, and Δ*nlpD* Δ*pbpG*) (Table S1). The localization of PBP7 in the Δ*amiABC*, Δ*envC*, and Δ*nlpD* mutants was also assessed through immunolabeling with purified antibodies against PBP7. The absence of amidases and their activator in the cells did not hinder the midcell localization of PBP7 (Figure S3). The above experiments suggest that, in contrast to PBP4, PBP7 does not support the amidase function. Instead, our work suggests that PBP7 may impact the assembly of FtsN within the divisome by regulating the synthesis of septal PG.

## 4 Material and Methods

### 4.1 Bacterial Strains and Culture Conditions

*E. coli* K12 strains used in this work are listed in Table S2. Strains were cultured in LB medium (10 g Tryptone (Duchefa), 5 g yeast extract (Fisher Bioreagents) and 10 g NaCl (Acros Organics) per liter) at 37°C, GB4 minimal medium (6.33 g K_2_HPO_4_ × 3H_2_O (VWR), 2.95 g KH_2_PO_4_ (Fisher Chemical), 1.05 g (NH_4_)_2_SO_4_ (Sigma‐Aldrich), 0.10 g MgSO_4_ × 7H_2_O (Roth), 0.28 mg FeSO_4_ × 7H_2_O (Sigma‐Aldrich), 7.1 mg Ca (NO_3_)_2_ × 4H_2_O (Sigma‐Aldrich), 4 mg thiamine (Sigma‐Aldrich), 2 μg uracil (Sigma‐Aldrich),  50 μg lysine, arginine, and glutamine (Sigma‐Aldrich), 20 g thymidine (Sigma‐Aldrich), and 4 g glucose (Roth, Karlsruhe), per liter, pH 7.0) at 28°C, and GB1 minimal medium at 28°C or 42°C. GB1 is the GB4 medium without, uracil, arginine and glutamine. Antibiotics, chloramphenicol (25 μg mL^−1^) (Sigma‐Aldrich), kanamycin (50 μg mL^−1^) (Sigma‐Aldrich), tetracycline (10 μg mL^−1^) (Sigma‐Aldrich), aztreonam (1 μg mL^−1^) (Sigma‐Aldrich), and ampicillin (100 μg mL^−1^) (Roth) were added to medium when necessary. Strains were cultured to steady state in rotating Erlenmeyer flasks in a water bath. To generate growth curves, the strains were cultured in 96‐well plates.

### 4.2 *Escherichia coli* Deletion Strains and Plasmids Construction

*Escherichia coli* deletion strains were constructed by λ‐Red recombination as described (Datsenko and Wanner 2000). The primers used for the construction of deletion strains were listed in Table S3. After PCR products size checking, purification, and DpnI digestion, the products were electroporated into cells harboring the plasmid pKD46. Recombinants were selected on LB plates containing 25 μg mL^−1^ chloramphenicol or 50 μg mL^−1^ kanamycin. The resistance gene in genome was removed by temperature‐sensitive plasmid pCP20 when necessary. Single mutants for amidases and their activator, as well as double mutants for PBP7 and one of the amidases or their activators, were generated using P1 transduction, as described previously (Thomason, Costantino, and Court 2007).

The plasmids used in our work were listed in Table S4, and the primers for plasmids construction were listed in Table S3. After PCR purification, PCR products were digested by DpnI and checked by DNA gel. PCR products for site‐mutation plasmids were directly transformed into DH5α competent cells for plasmids storing and checking. Gibson et al. (2009) assembly was employed for the construction of new plasmids and the assembled products were directly transformed into DH5α competent cells for plasmids storing and checking.

### 4.3 Immunolabeling

The strains used for immunolabeling were cultured in GB1 or GB4 medium to steady state at 28°C, cells were fixed by 2.8% formaldehyde, and 0.004% glutaraldehyde and continued to incubate for 15 min. The immunolabeling experiments were carried out as described before (Buddelmeijer, Aarsman, and den Blaauwen 2013). The rabbit polyclonal PBP7 antibody (1:200), FtsN antibody (1:500) (Aarsman et al. 2005), FtsZ antibody (1:500) (Aarsman et al. 2005), PBP3 antibody (1:100) (Marrec‐Fairley et al. 2000), and secondary antibody Cy3‐AffiniPure Donkey Anti‐Rabbit IgG (1:300) (Jackson Immunochemistry) were used in this work. The immunolabeled cells were immobilized on 1% agarose and pictured with the BX‐60 fluorescence microscope (Olympus) equipped with a Hamamatsu ORCAFlash‐4.0LT CMOS camera (Naka‐ku) fluorescence microscope through a 100x/N.A. 1.35 oil objective. Images were taken using the program ImageJ (http://imagej.nih.gov/ij/) with MicroManager (https://www. micro‐manager.org). An mCherry fluorescence filter (excitation at 560 ± 40 nm and emission at 630 ± 75 nm) was used.

### 4.4 Immunoblotting

Samples were separated by SDS‐PAGE and transferred onto a nitrocellulose in a semi‐dry transfer manner as described previously (Mertens and den Blaauwen 2022). The membranes were blocked with 5% skimmed milk in TBS solution for 1 h and then incubated overnight with appropriate primary antibodies (1:2000 for α‐PBP7) at 4°C. Membranes were washed three times with TBST solution and incubated with secondary antibodies (1:5000) tagged with horseradish peroxidase (HRP) (SAB3700863, Sigma‐Aldrich) for 1 h at room temperature. The chemical signal of HPR was detected by ECL Prime detection substrate (32109, Thermo Fisher Scientific).

### 4.5 Bocillin‐Binding Assays

Strains were cultured In LB medium overnight at 37°C. The second day, overnight mediums were diluted 1:1000 in fresh LB medium and grown until an OD_600_ ≈ 0.3. Cells were collected by centrifugation (8000×*g* for 2 min at room temperature). After two washes with 1 mL of PBS, the pellet was resuspended in 50 μL PBS containing 5 μg mL^−1^ Bocillin‐FL (B13233, Thermo Fisher Scientific) and incubated for 10 min. The pellets were washed twice with 100 μL PBS and then resuspended in 100 μL PBS to which 20 μL 5X protein loading buffer (250 mM Tris buffer at pH 8.3 10% SDS, 500 mM DTT, and 50% Glycerol) was added. The samples were heated for 10 min at 99°C to denature the protein samples and 10 μL sample was loaded on a 10% SDS‐PAGE gel. The gel was scanned using a LICOR Odyssey M Imager (LI‐COR, United States of America) at 520 nm.

### 4.6 HADA Labelling

The strains in the exponential growth stage were harvested by centrifugation at 8000×*g* for 2 min and then resuspended in pre‐warmed LB medium, supplemented with 250 μM HADA, for a 10‐min incubation at 37°C. Each sample was separated into two aliquots. One aliquot was collected by centrifugation (8000×*g* for 2 min) and fixed in 1 mL 70% ethanol for 10 min. After two times washing with 1 mL PBS, samples were ready for imaging. The other aliquot was washed twice with pre‐warmed LB medium at 8000×*g* for 2 min and resuspended in pre‐warmed LB medium. After 20 min additional cultivation at 37°C, the cells were fixed in 1 mL 70% ethanol for 10 min. After two times washing with 1 mL PBS, samples were immobilized on 1% agarose (Koppelman et al. 2004) and imaged as described above. The filter used was U‐MWU (Olympus, excitation at 330–385 nm and emission from 420 nm long pass).

### 4.7 Image Analysis

The phase‐contrast and fluorescence images were captured with the ImageJ 1.53f program (http://imagej.nih.gov/ij/) and merged into hyperstacks. The Coli‐Inspector project file, in conjunction with the ObjectJ‐1.05n plugin (https://sils.fnwi.uva.nl/bcb/objectj/), was utilized for the analysis of cellular morphology, fluorescence‐related properties, and cell age, as previously described (Vischer et al. 2015). Cell length, cell diameter, cell volume, and constricting cells, among other parameters, can be measured. The fluorescence intensity corresponding to the local fluorescence of an individual cell can be tracked. Cells were sorted based on their length, and the fluorescence intensity of each individual cell's local fluorescence was depicted in demographs, with each cell represented by a one‐pixel line.

### 4.8 ΔpbpG and Amidases/Regulators Imaging

Overnight cultures were adjusted to an initial OD at 600 nm of 0.01 in LB, and the cells were incubated at 37°C until the OD reached approximately 0.2. A volume of 500 μL of the culture was fixed by adding 2.4% formaldehyde and 0.04% glutaraldehyde. Fixed cells were inoculated on agarose pads prepared with 1.5% agarose in PBS and set in Gene Frames (Thermo Scientific). Imaging was conducted using a Zeiss AxioObserver equipped with a Plan‐Apochromat 100×/Oil Ph3 objective, and illumination was provided by HXP 120 V for phase‐contrast images. For phenotype analysis in Table S1, we used MicrobeJ plugin for Fiji 600 (Ducret, Quardokus, and Brun 2016).

## Author Contributions

**Xinwei Liu:** conceptualization, data curation, formal analysis, funding acquisition, investigation, writing – original draft, methodology. **Gabriela Boelter:** data curation, formal analysis, investigation, writing – review and editing. **Waldemar Vollmer:** conceptualization, formal analysis, funding acquisition, resources, writing – review and editing. **Manuel Banzhaf:** conceptualization, formal analysis, funding acquisition, resources, writing – review and editing. **Tanneke den Blaauwen:** conceptualization, data curation, formal analysis, methodology, project administration, resources, supervision, visualization, writing – original draft, writing – review and editing.

## Ethics Statement

The authors have nothing to report.

## Conflicts of Interest

The authors declare no conflicts of interest.

## Supporting information


SUPPORTING INFORMATION S1.

## Data Availability

All data are provided in this manuscript and in the supporting data.
