# Supplementary material for: Peptidoglycan Endopeptidase PBP7 Facilitates the Recruitment of FtsN to the Divisome and Promotes Peptidoglycan Synthesis in Escherichia coli
Source: Mol Microbiol. 2024 Sep 30;122(5):743–56. doi: 10.1111/mmi.15321 (PMC11586513; doi:10.1111/mmi.15321)
Supplement: Supplementary file 1 — SUPPORTING INFORMATION S1. [file MMI-122-743-s001.docx]

**Supplementary information**

**Peptidoglycan endopeptidase PBP7 facilitates the recruitment of FtsN to the divisome and promotes peptidoglycan synthesis in *Escherichia coli***

Xinwei Liu^1^, Gabriela Boelter^4^, Waldemar Vollmer^2,3^, Manuel Banzhaf^2,4^ and Tanneke den Blaauwen^1^*

^1^ Bacterial Cell Biology, Swammerdam Institute for Life Sciences, Faculty of Science, University of Amsterdam, Amsterdam, The Netherlands.

^2^ Centre for Bacterial Cell Biology, Biosciences Institute, Newcastle University, Newcastle upon Tyne, United Kingdom.

^3^ Institute for Molecular Bioscience, The University of Queensland, Brisbane, QLD, Australia.

^4^ Institute of Microbiology & Infection and School of Biosciences, University of Birmingham, Edgbaston, Birmingham, United Kingdom

* Correspondence: Tanneke den Blaauwen, [t.denblaauwen@kpnmail.nl](mailto:t.denblaauwen@kpnmail.nl)


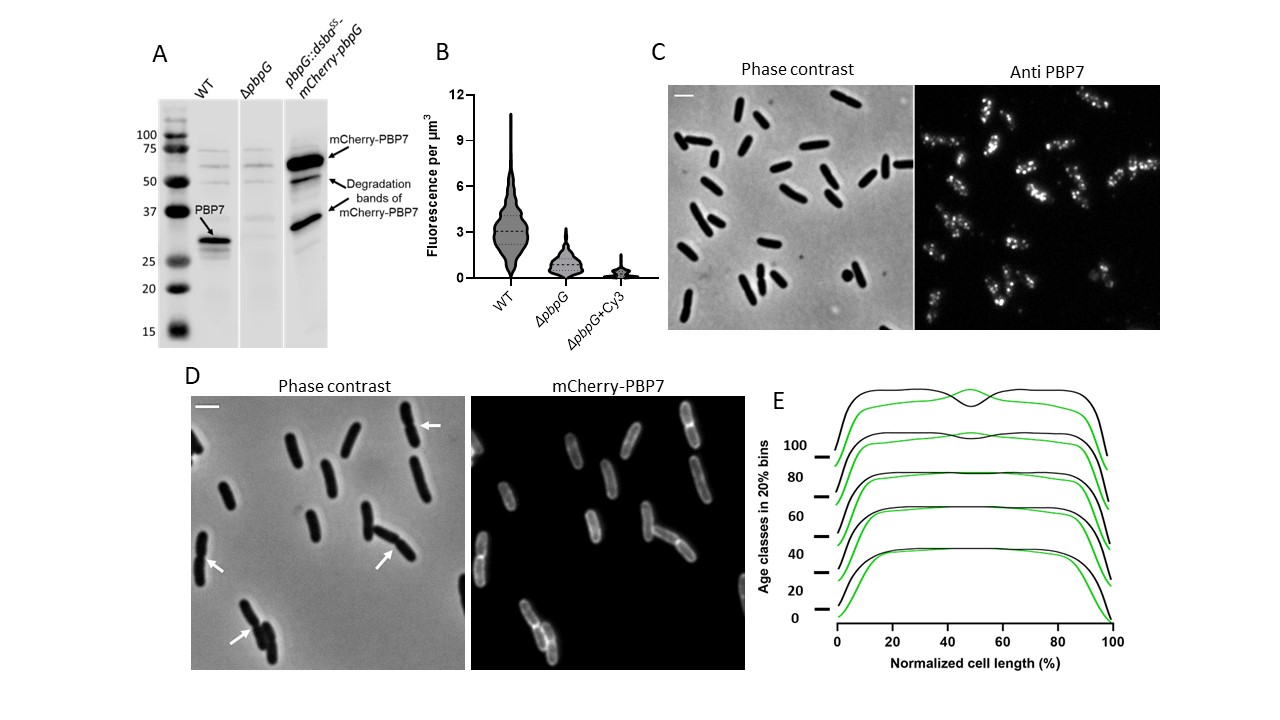


Figure S1. PBP7 localization in fixed cells and mCherry-PBP7 localization in live cells. A. Antibody specificity for PBP7 and stability of mCherry-PBP7 was assessed by Western blot. The bands for PBP7, mCherry-PBP7 and mCherry-PBP7 degradation products are indicated by black arrows. B. The indicated strains cultured in LB medium were fixed by FA/GA and immunolabeled with purified antibody for PBP7. As a negative control, only the secondary antibody Cy3-AffiniPure Donkey Anti-Rabbit IgG was used to immunolabel the Δ*pbpG* mutant. C Images of the immunolabelled wild-type cells with purified antibodies for PBP7. The left panel is a phase contrast image, and the right panel is the corresponding fluorescence image. The scale bar equals 2 μm. D. Images of cells expressing DsbA^ss^-mCherry-PBP7 grown in GB4 medium without IPTG. The left panel is a phase contrast image of live cells expressing mCherry-PBP7, and the right panel is the corresponding fluorescence image. The constricting cells are indicated by white arrows. The scale bar equals 2 μm. E. The diameter (black lines) of *pbpG*::*DsbA^ss^-mCherry-pbpG* cells and mCherry fluorescence (green lines) profiles along the normalized cell length were plotted in 20% age class bins. More than 2000 cells were included.


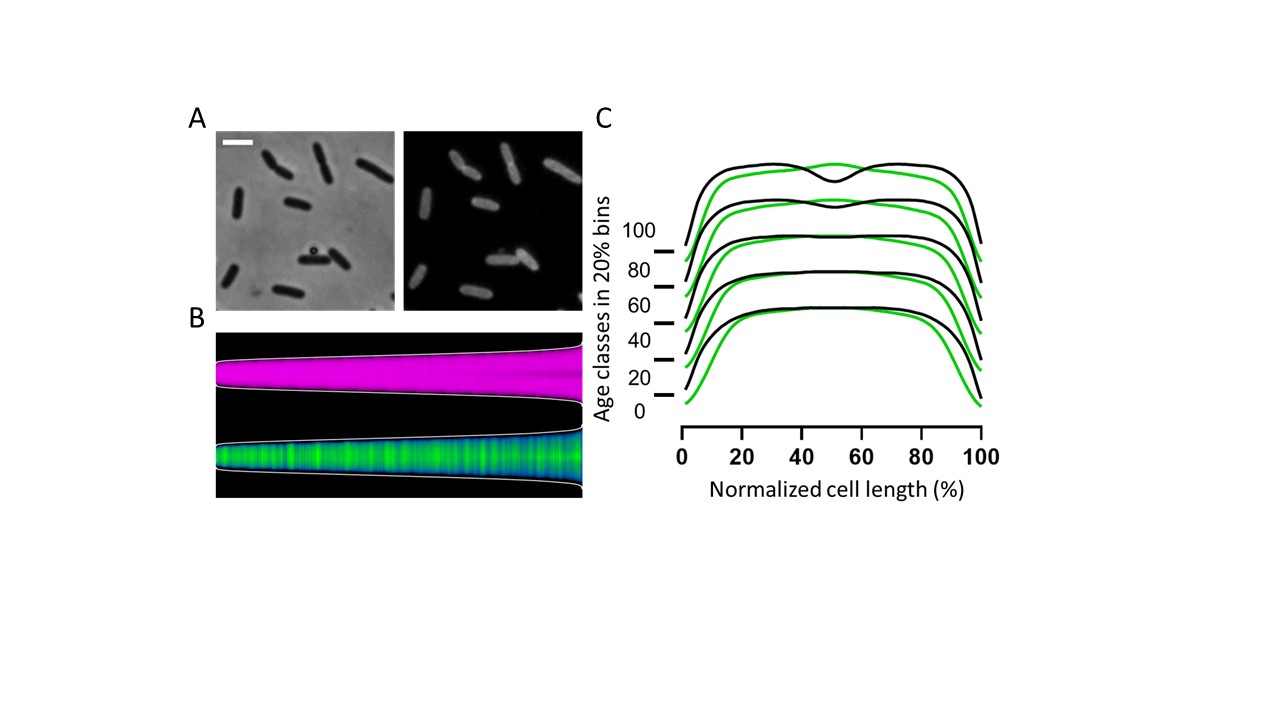


Figure S2. Fluorescence of bodipy in fixed wild-type cells. A. The left panel is a phase contrast image of cells stained with bodipy, and the right panel is the corresponding fluorescence image. The scale bar equals 2 μm. B. Demographs of wild-type cell diameter (magenta) and bodipy fluorescence profiles (green) were sorted according to cell length (ascending from left to right). C. The wild-type cell diameter (black lines) and bodipy fluorescence (green lines) profiles along the normalized cell length were plotted in 20% age class bins. More than 8000 cells were included.


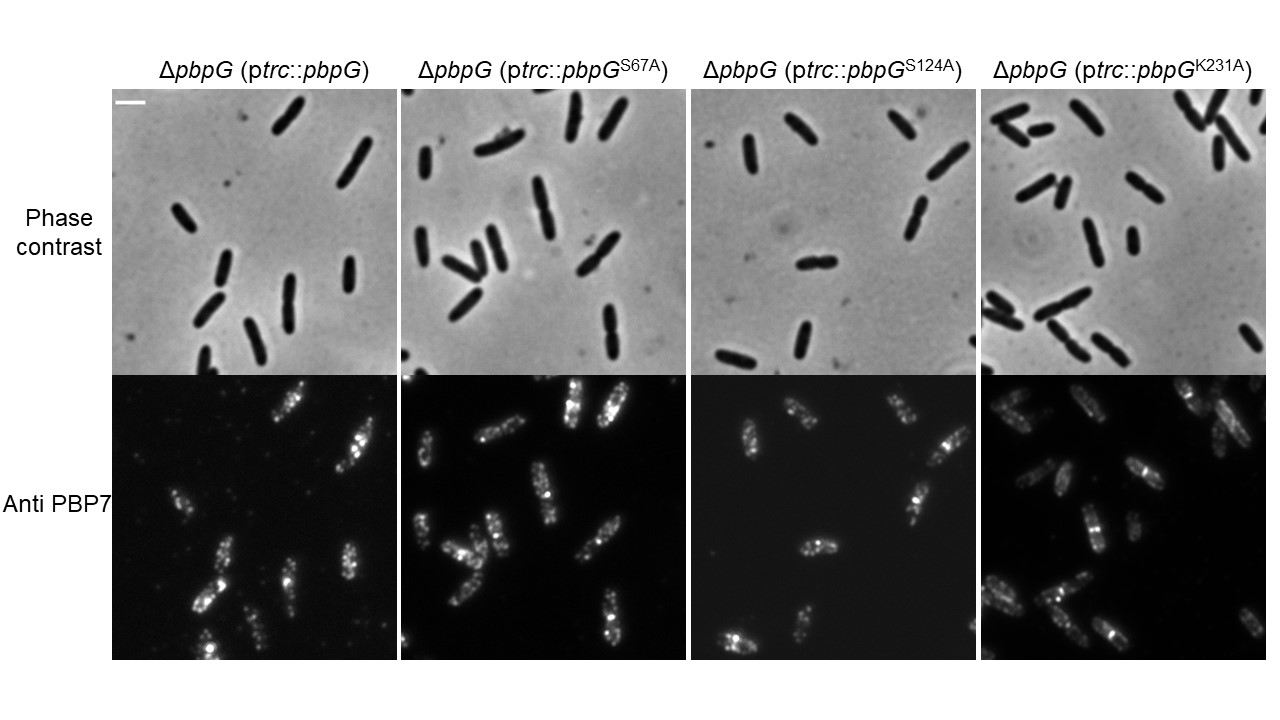


Figure S3. The localization of wild-type PBP7 and its three inactive variants in Δ*pbpG* cells. The Δ*pbpG* strain carrying a vector expressing PBP7, PBP7^S67A^, PBP7^S124A^ or PBP7^K231A^ were cultured in GB4 medium without IPTG at 28°C. The cells were fixed and immunolabeled with antibodies against PBP7. The phase contrast image and corresponding fluorescence image are present for each sample. The scale bar equals 2 μm.


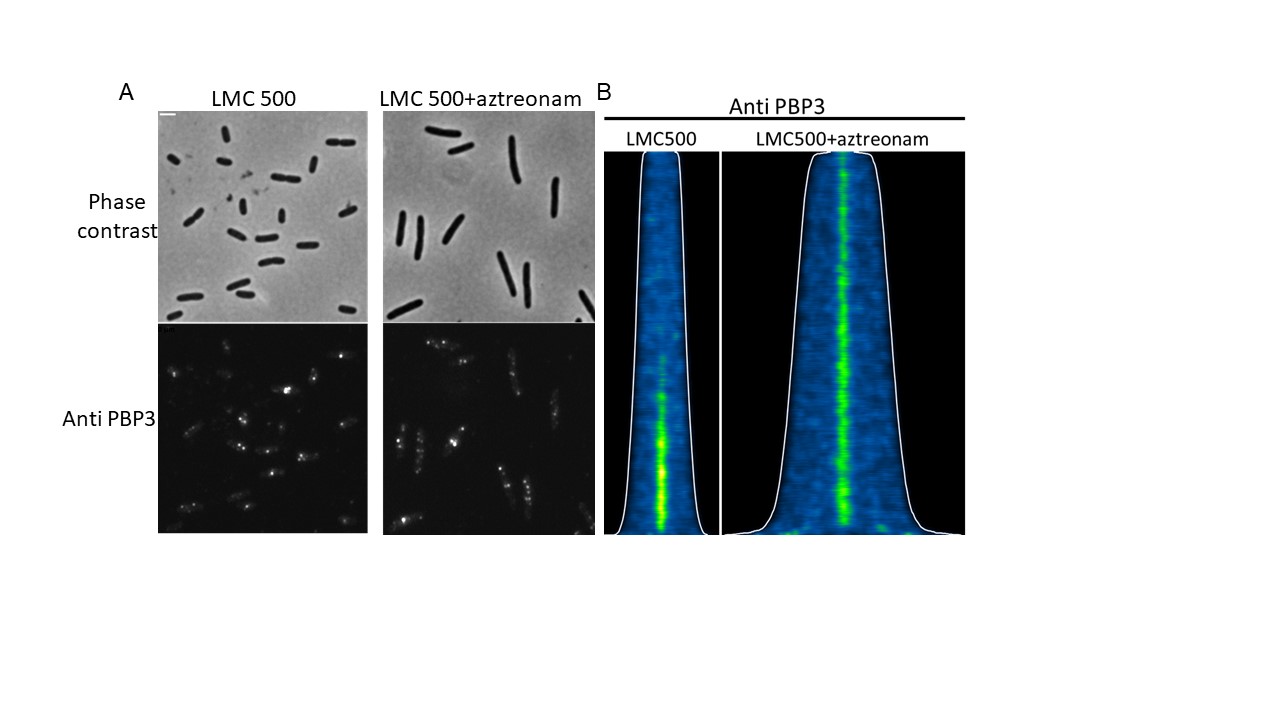


Figure S4. Subcellular localization of PBP3. A. LMC 500 grown to steady state in GB1 medium at 28°C, subsequently cultured in the same medium in the absence and presence of 1 µg/ml aztreonam for an additional mass doubling time. The cells were fixed and immunolabeled with antibodies against PBP3. The phase-contrast image and corresponding fluorescence image are present for each sample. The scale bar equals 2 μm. B. The demographs sort cells according to their length. The analyzed number of cells were 3070 and 2979 for cells cultivated in GB1 medium and GB1 medium with aztreonam, respectively.


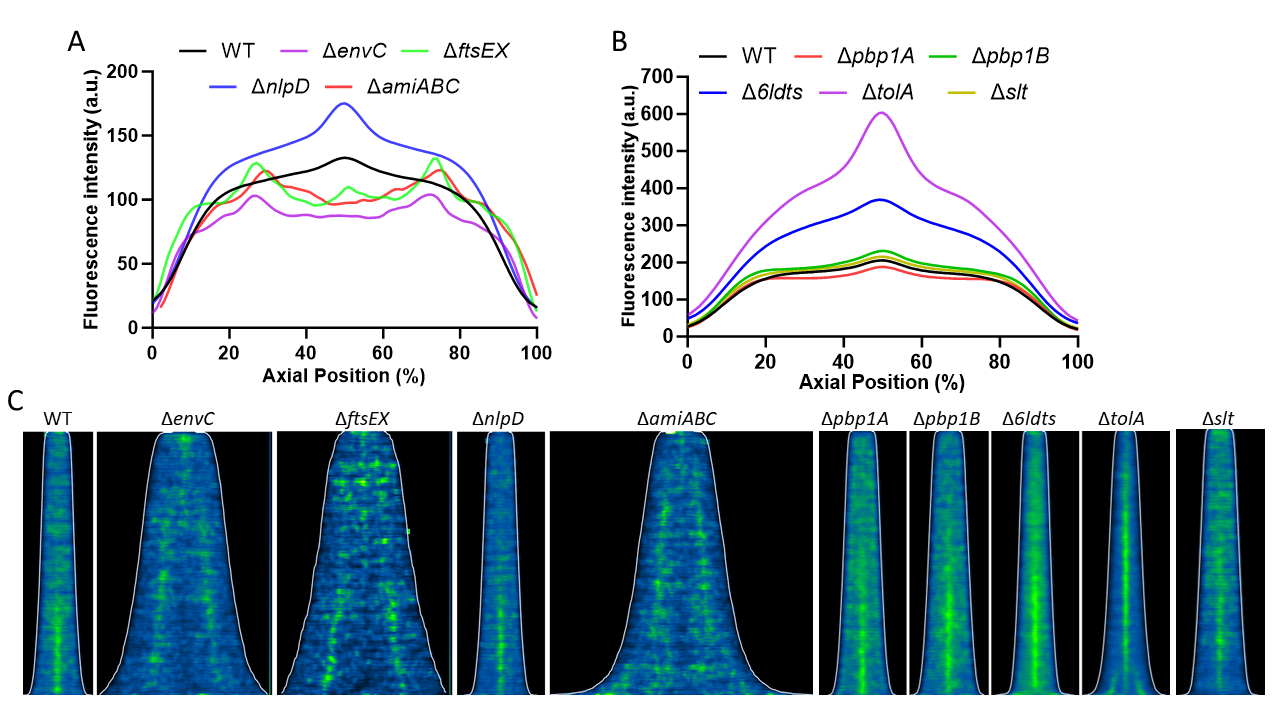


Figure S5. Subcellular localization of PBP7 in various mutant strains. Mutants and their parental strain wild-type BW25113 were grown to steady state in GB4 medium at 28℃, fixed by FA/GA and immunolabelled with antibodies against PBP7. A. The fluorescence intensity of PBP7 was normalized along the axial position of the cells. The cell number analysed were 6131 for wild-type cells, 1283 for Δ*envC,* 410 for Δ*ftsEX,* 4276 for Δ*nlpD* and 1199 for Δ*amiABC.* B. The fluorescence intensity of PBP7 was normalized along the axial position of the cells. The cell number analysed were 3268 for wild-type cells, 5320 for Δ*pbp1A,* 4021 for Δ*pbp1B, 5903 for* Δ*6ldts,* 5462 for Δ*tolA* and 2799 for Δ*slt*. C. The demographs of strains are sorted according to their cell length. The brightness and contrast of the demographs were adjusted to enhance the visibility of PBP7 localization and do therefore not reflect the amount of PBP7 in samples.


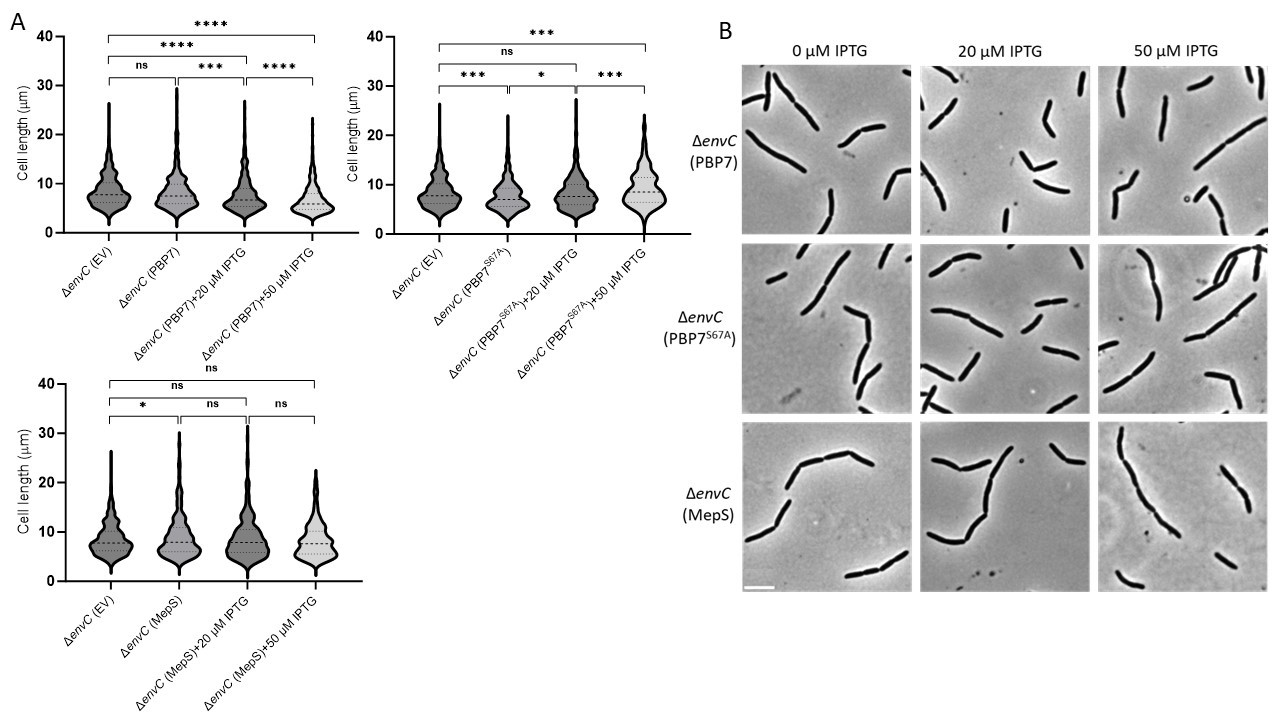


Figure S6. The overproduction of PBP7 reduces the cell length of the Δ*envC* mutant. A. The cell length of Δ*envC* mutants carrying an empty vector (EV) or vectors expressing PBP7, PBP7^S67A^ or MepS was measured at different concentrations of IPTG in GB1 medium at 28℃. An unpaired *t*-test has been used for statistical analysis, with *P* > 0.05 = ns, *P* ≤ 0.05 = *, *P* ≤ 0.01 = **, *P* ≤ 0.001 = *** *P* ≤ 0.0001= ****. The means in the violin bar graphs were quantified from more than 300 cells for each sample. B. The phase contrast images of Δ*envC* mutants carrying an empty vector (EV) or a vector expressing PBP7, PBP7^S67A^ or MepS at different concentrations of IPTG in GB1 medium at 28℃. The scale bar equals 5 µm.


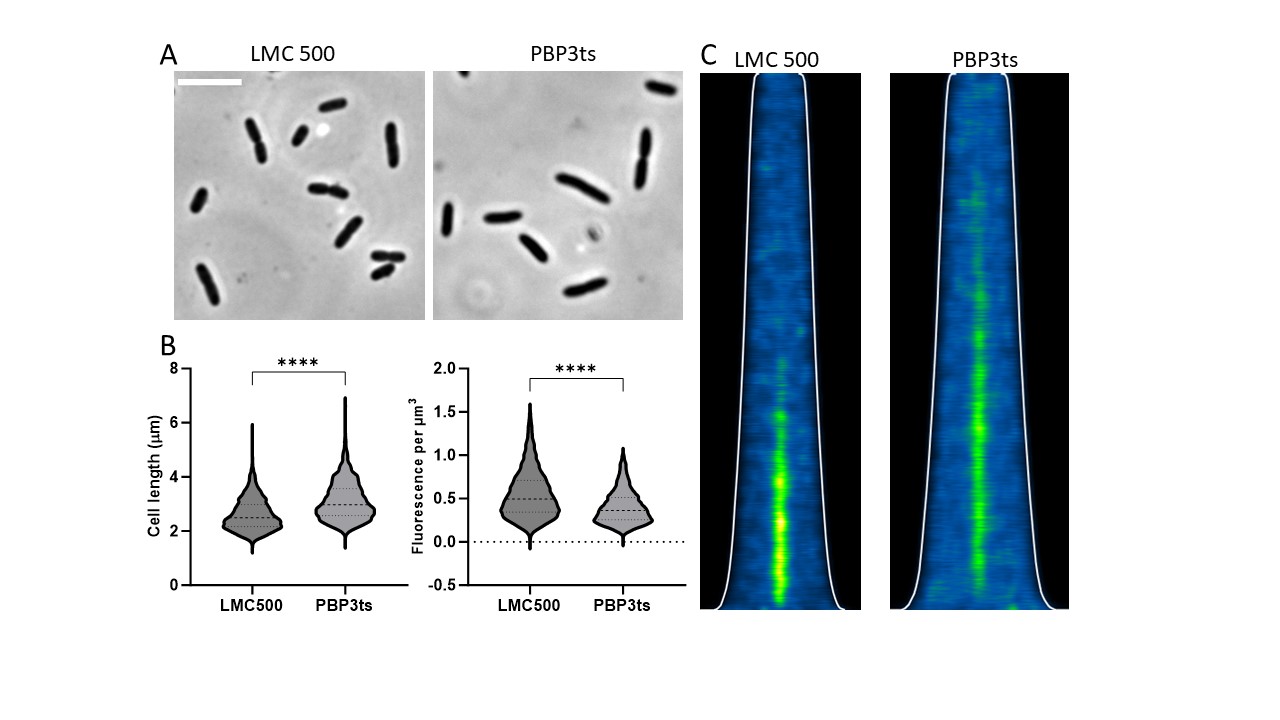


Figure S7. Morphological analysis and midcell localization of PBP3 in LMC500 and PBP3ts strains. Cells were grown to steady state in GB1 medium at 28℃, fixed by FA/GA and subjected to immunolabeling using antibodies that recognize PBP3. A. The Phase contrast images of LMC500 and PBP3ts. B. The cell length and the concentration of PBP3 in LMC500 and PBP3ts strains were quantified in fluorescence units per μm^3^. An unpaired *t*-test has been used for statistical analysis, with *P* ≤ 0.0001= ****. The means in the violin bar graphs were quantified from more than 3000 cells for each sample. C. In the demographs, the cells are sorted based on their length. The brightness and contrast of the demographs were adjusted to enhance the visibility of PBP3 localization and do therefore not reflect the amount of PBP3 in samples. The scale bar equals 5 µm.

**Table S1. Phenotypes of *pbpG*, amidases and regulators mutants**

1 All cells were considered single cells independent of the number of segments.

2 Total length means cumulative length of all cells measured.

3 Refers to the total length/number of cells.

4 Septa are considered as any membrane constrictions or completed membrane septa in cell chains.

5 Length/septum indicates the total length/total number of septa. It shows the frequency at which septa are detected. The number is much lower in chaining cells because septa persist for an abnormally long time.

6 The number of cell segments refers to the number of cells plus the number of septa. The “length/segment” is the total length/total number of segments. In normal (nonchaining) cells, this measurement is similar to the average cell length (pole-pole distance), but the value is smaller because predivisional cells contain two segments and are counted as two cells instead of one (i.e., some pole-to-septa measurements are taken into account, as well as pole-pole measurements). In chaining cells, the length/segment measurement refers mainly to the distance between adjacent septa.

7 Cells with more than one septum are considered chaining cells.

8 Percentage of chaining cells related to the total no. of cells.

| **Relevant genotype** | **No. of cells^1^** | **Total length (µm)^2^** | **Avg length (µm)^3^** | **Total width (µm)** | **Avg width (µm)** | **Total no. of septa^4^** | **Length/ septum (µm)^5^** | **Length/**  **segment (µm)^6^** | **No. of septa/cell** | **No. of cells chaining^7^** | **Percentage of cells chaining^8^** |
| --- | --- | --- | --- | --- | --- | --- | --- | --- | --- | --- | --- |
| **WT** | 126 | 586.6 | 4.7 | 128.9 | 1.0 | 95 | 6.2 | 2.7 | 0.8 | 0.0 | 0.0 |
| **∆*pbpG*** | 365 | 1419.6 | 3.9 | 384.3 | 1.1 | 147 | 9.7 | 2.8 | 0.4 | 1 | 0.3 |
| **∆*amiA*** | 327 | 1643.1 | 5.0 | 342.1 | 1.0 | 224 | 7.3 | 3.0 | 0.7 | 4 | 1.2 |
| **∆*amiB*** | 203 | 967.8 | 4.8 | 210.6 | 1.0 | 99 | 9.8 | 3.2 | 0.5 | 1 | 0.5 |
| **∆*amiC*** | 211 | 986.4 | 4.7 | 219.8 | 1.0 | 112 | 8.8 | 3.1 | 0.5 | 1 | 0.5 |
| **∆*envC*** | 298 | 2260.2 | 7.6 | 328.7 | 1.1 | 439 | 5.1 | 3.1 | 1.5 | 80 | 26.8 |
| **∆*nlpD*** | 228 | 1045.6 | 4.6 | 235.5 | 1.0 | 95 | 11.0 | 3.2 | 0.4 | 1 | 0.4 |
| **∆*pbpG* ∆*amiA*** | 284 | 1304.7 | 4.6 | 298.8 | 0.8 | 110 | 11.9 | 3.3 | 0.4 | 2 | 0.7 |
| **∆*pbpG* ∆*amiB*** | 278 | 1122.9 | 4.0 | 295.7 | 1.1 | 120 | 9.4 | 2.8 | 0.4 | 4 | 1.4 |
| **∆*pbpG* ∆*amiC*** | 240 | 999.2 | 4.2 | 256.2 | 1.1 | 111 | 9.0 | 2.8 | 0.5 | 5 | 2.1 |
| **∆*pbpG* ∆*envC*** | 329 | 2601.3 | 7.9 | 362.1 | 1.1 | 454 | 5.7 | 3.3 | 1.4 | 95 | 28.9 |
| **∆*pbpG* ∆*nlpD*** | 259 | 1179.3 | 4.6 | 323.0 | 1.1 | 128 | 9.2 | 3.0 | 0.5 | 0 | 0.0 |
| **∆*pbpG* ∆*dacB*** | 292 | 1135.6 | 3.9 | 289.9 | 0.9 | 127 | 8.9 | 2.7 | 0.4 | 0 | 0.0 |

**Table S2. Strains in this work**

| **Strain** | **Mutation** | **Genotype** | **Source** |
| --- | --- | --- | --- |
| LMC500 | Wild-type | F^-^, *araD139, ∆(argF-lac)U169, deoC1, flbB5301, lysA1, ptsF25, rbsR, relA1, rpsL150* | (Taschner *et al.,* 1988) |
|  | Δ*pbpG* | Δ*pbpG::kan^R^* | This work |
|  | Δ*amiA* | Δ*amiA::kan^R^* | This work |
|  | Δ*amiB* | Δ*amiB::kan^R^* | This work |
|  | Δ*amiC* | Δ*amiC::kan^R^* | This work |
|  | Δ*envC* | Δ*envC::kan^R^* | This work |
|  | Δ*nlpD* | Δ*nlpD::kan^R^* | This work |
|  | Δ*pbpG* Δ*amiA* | Δ*amiA* Δ*pbpG::kan^R^* | This work |
|  | Δ*pbpG* Δ*amiB* | Δ*amiB* Δ*pbpG::kan^R^* | This work |
|  | Δ*pbpG* Δ*amiC* | Δ*amiC* Δ*pbpG::kan^R^* | This work |
|  | Δ*pbpG* Δ*envC* | Δ*envC* Δ*pbpG::kan^R^* | This work |
|  | Δ*pbpG* Δ*nlpD* | Δ*nlpD* Δ*pbpG::kan^R^* | This work |
|  | *JOE565* Δ*FtsN + FtsN* | Δ*ftsN::Kan^R^, with plasmid pJC83 expressing FtsN from arabinose inducible pBad33 vector* | (Chen & Beckwith, 2001) |
|  | *PBP3ts* | *ftsI 2158* | (Taschner *et al.,*1988) |
| BW25113 | Wild-type | *F^-^, Δ(araD-araB)567, lacZ4787(del)::rrnB-3, LAM-, rph-1, Δ(rhaD-rhaB)568, hsdR514* | (Datsenko & Wanner, 2000) |
|  | Δ*pbpG* | Δ*pbpG::kan^R^* | This work |
|  | *pbpG::DsbA^ss^-mCherry-pbpG* | *pbpG::dsbA^ss^-mcherry-pbpG* | This work |
|  | Δ*tolA* | Δ*tolA::kan^R^* | (Baba *et al.,* 2006) |
|  | ∆*pbp1A* | ∆*pbp1A:: tet^R^* | (Baba *et al.,* 2006) |
|  | ∆*pbp1B* | ∆*pbp1B:: tet^R^* | (Baba *et al.,* 2006) |
|  | ∆*envC* | Δ*envC::kan^R^* | (Baba *et al.,* 2006) |
|  | ∆*FtsEX* | Δ*ftsEX::cat^R^* | (Verheul *et al.,* 2022) |
|  | ∆*nlpD* | Δ*nlpD::kan^R^* | (Baba *et al.,* 2006) |
|  | ∆*amiABC* | Δ*amiABC::cat^R^, kan^R^* | (Heidrich *et al.,* 2001) |
|  | ∆*6ldts* | ∆*6ldts::kan^R^* | (Kuru *et al.,* 2017) |
|  | ∆*slt* | ∆*slt::kan^R^* | (Baba *et al.,* 2006) |
| DH5α | Storage strain | F^-^ *endA1 glnV44 thi-1 recA1* ϕ80Δ*lacZ*∆M15, λ^-^ | Invitrogen |

**Table S3. Primers in this work**

| **Purposes** | **Name** | **Sequence 5’-3’** |
| --- | --- | --- |
| Knock out PBP7 in genome | XW KO PBP7-F | ccggcggtgcgcaacccgtgcgcgtgaaccactatctgagtgtaggctggagctgcttc |
|  | XW KO PBP7-R | aaaaattacggatggcagagtatcgccatccgaattcacatgggaattagccatggtcc |
| Fuse PBP7 in genome with mCherry | XW mCherry-pbp7-1 | ctgctcgccctaaccgttat |
|  | XW mCherry-pbp7-2 | gaagcagctccagcctacacgatgagcattcagatagtggttcacg |
|  | XW mCherry-pbp7-3 | catcgtgtaggctggagctgcttc |
|  | XW mCherry-pbp7-4 | caccgtgcagtcgatgataagctgtcaaacatgggaattagccatggtcc |
|  | XW mCherry-pbp7-5 | gtttgacagcttatcatcgactgcacggtg |
|  | XW mCherry-pbp7-6 | gttttctgaagacatcagcgccagcaacag |
| PBP7 genome check | XW G-*pbpG* C-F | catttcctcacgattctcctttgac |
|  | XW G-*pbpG* C-R | gtcatttgtataaagcaccggagac |
| pXWL-PBP7S1 Construction | XW *pbpG* S67A-F | atctggtgcgtccgattgcggctatcagcaaattaatgacc |
|  | XW *pbpG* S67A-R | ggtcattaatttgctgatagccgcaatcggacgcaccagat |
| pXWL-PBP7S2 Construction | XW *pbpG* K70A-F | gtccgattgcgtctatcagcgcattaatgaccgcgatggttg |
|  | XW *pbpG* K70A-R | caaccatcgcggtcattaatgcgctgatagacgcaatcggac |
| pXWL-PBP7S3 Construction | XW *pbpG* S124A-F | tgttgctggcgctgatgtctgcagaaaaccgcgcggcggca |
|  | XW *pbpG* S124A-R | tgccgccgcgcggttttctgcagacatcagcgccagcaaca |
| pXWL-PBP7S4 Construction | XW *pbpG* K231A-F | actggaatattcagttaaccgcaaccggctttaccaatgcgg |
|  | XW *pbpG* K231-R | ccgcattggtaaagccggttgcggttaactgaatattccagt |
| pXWL001- PBP7^ΔCα^ | XW 37 | ggtaaagtgatgcctgtgccgtaaaagcttgggcccgaacaaaaac |
|  | XW 38 | cggcacaggcatcactttacc |
| PBP7 plasmid check | XW P-*pbpG* C-F | gcactcccgttctggataatg |
|  | XW P-*pbpG* C-F | ttatcagaccgcttctgcg |
| pXWL018-dsba-mCherry-PBP7-C-helix | XW 61 | ggtaaagtgatgcctgtgccgg |
|  | XW 62 | acaggcatcactttacccttgtacagctcgtccatgccgc |
| pXWL027-mCherry-FtsN SPOR domain | XW 84 | aaagacgaacgccgctggatgg |
|  | XW 85 | ccatccagcggcgttcgtctttcttgtacagctcgtccatgccg |
|  | XW 92 | aaagaagcttggctgttttggcggatgagagaag |
|  | XW 93 | cttctctcatccgccaaaacagccaagcttcttttcaacccccggcggcgagcc |
| pXWL046-MepS | XW 123 | tttcacacaggaaacagaccatggtcaaatctcaaccgattttgagatatatct |
|  | XW 124 | aacagccaagcttcttttcattagctgcggctgagaaccc |
|  | XW 63 | aaagaagcttggctgttttggcg |
|  | XW 63 | ggtctgtttcctgtgtgaaattgt |

**Table S4. Plasmids in this work**

| **Name** | **Characteristics** | **Source** |
| --- | --- | --- |
| PKD3 | contain the FRT-CAM-FRT cassette for gene inactivation | (Wanner, 2000) |
| PKD4 | contain the FRT-KAN-FRT cassette for gene inactivation | (Wanner, 2000) |
| PKD46 | Contain λ-phage red recombinase proteins induced by arabinose | (Wanner, 2000) |
| pCP20 | Thermal induction of FLP synthesis | (Wanner, 2000) |
| pXWL027 | pSAV057-DsbA^SS^-mCherry-SPOR^FtsN^ | This work |
| pSAV057 | Basic expression vector, p*trc* promoter, p15 origin and cat^R^ | (Alexeeva *et al.,* 2010) |
| pXWL058 | pSAV057-DsbA^SS^-HA-MltD | This work |
| PSF001 | pSAV057-PBP7 | This work |
| pXWL-PBP7S1 | pSAV057-PBP7^S67A^ | This work |
| pXWL-PBP7S2 | pSAV057-PBP7^K70A^ | This work |
| pXWL-PBP7S3 | pSAV057-PBP7^S124A^ | This work |
| pXWL-PBP7S4 | pSAV057-PBP7^K231A^ | This work |
| pXWL001 | pSAV057-PBP7^ΔCα^ | This work |
| pXWL018 | pSAV057-DsbA^ss^-mCherry-C-α-helix^PBP7^ | This work |
| pSAV057-DsbA^ss^-mCherry | psav057-DsbA^ss^-mCherry | This work |

**Reference**

Alexeeva S, Gadella TW, Jr., Verheul J, Verhoeven GS, den Blaauwen T. 2010. Direct interactions of early and late assembling division proteins in *Escherichia coli* cells resolved by FRET. *Mol Microbiol* 77: 384-98

Baba T, Ara T, Hasegawa M, Takai Y, Okumura Y, *et al*. 2006. Construction of *Escherichia coli* K-12 in-frame, single-gene knockout mutants: the Keio collection. *Mol Syst Biol* 2: 2006.0008

Chen JC, Beckwith J. 2001. FtsQ, FtsL and FtsI require FtsK, but not FtsN, for co-localization with FtsZ during *Escherichia coli* cell division. *Mol Microbiol* 42: 395-413

Datsenko KA, Wanner BL. 2000. One-step inactivation of chromosomal genes in *Escherichia coli* K-12 using PCR products. *Proc Natl Acad Sci U S* *A* 97: 6640-45

Heidrich C, Templin MF, Ursinus A, Merdanovic M, Berger J, *et al*. 2001. Involvement of N-acetylmuramyl-l-alanine amidases in cell separation and antibiotic-induced autolysis of *Escherichia coli*. *Mol Microbiol* 41: 167-78

Kuru E, Lambert C, Rittichier J, Till R, Ducret A, *et al*. 2017. Fluorescent D-amino-acids reveal bi-cellular cell wall modifications important for *Bdellovibrio bacteriovorus* predation. *Nat Microbiol* 2: 1648-57

Taschner PE, Huls PG, Pas E, Woldringh CL. 1988. Division behavior and shape changes in isogenic *ftsZ, ftsQ, ftsA, pbpB,* and *ftsE* cell division mutants of *Escherichia coli* during temperature shift experiments. *J Bacteriol* 170: 1533-40

Verheul J, Lodge A, Yau HCL, Liu X, Boelter G, et al. 2022. Early midcell localization of *Escherichia coli* PBP4 supports the function of peptidoglycan amidases. *PLoS Genet* 18: e1010222
